# Untargeted Blubber Metabolomics Reveals Biochemical Signatures Associated with Physiological Status in Live, Free-Ranging Bottlenose Dolphins

**DOI:** 10.3390/metabo16070473

**Published:** 2026-07-06

**Authors:** Makayla A. Guinn, Dara N. Orbach, Hussain Abdulla

**Affiliations:** 1 Department of Life Sciences, Texas A&M University-Corpus Christi, Corpus Christi, TX 78412, USA; dnorbach@gmail.com; 2 Department of Physical and Environmental Science, Texas A&M University-Corpus Christi, Corpus Christi, TX 78412, USA; hussain.abdulla@tamucc.edu

**Keywords:** blubber, cetacean, health, human impacts, liquid chromatography, mass spectrometry, metabolome, physiology

## Abstract

**Highlights:**

**What are the main findings?**

- Seasonal differences in dolphin blubber metabolites suggest enhanced lipid quality and immune regulation in spring and increased metabolic flexibility and stress-associated responses in summer.
- Blubber metabolite profiles varied across nearby sites, with two locations exhibiting metabolite profiles consistent with lipid-based energy metabolism and one showing protein turnover pathways suggestive of energetic imbalance.

**What are the implications of the main findings?**

- Blubber metabolites extracted from biopsy samples of live dolphins serve as integrative biomarkers of physiological adaptations that closely reflect habitat conditions, prey availability, and stressors.
- This study represents the first application of blubber metabolomics in free-ranging bottlenose dolphins, highlighting its potential for broad use in monitoring population health and resilience across other marine mammal species.

**Abstract:**

**Background/Objectives:** Dolphins inhabiting coastlines can be influenced by anthropogenic factors. As biochemical changes accumulate in blubber over weeks to months, blubber metabolites may be informative biomarkers of molecular adaptations to environmental changes. **Methods**: We investigated the blubber metabolomic signatures of live free-ranging bottlenose dolphins for the first time. This exploratory study analyzed blubber samples from 35 common bottlenose dolphins (*Tursiops truncatus*) in South Texas waters using untargeted ultra-high-performance liquid chromatography-Orbitrap metabolomics. **Results**: Blubber samples exhibited distinct temporal and spatial metabolic patterns. Pathway enrichment analyses comparing detected metabolites (*n* = 2777) revealed that dolphins sampled in the spring had enhanced lipid quality and immune regulation, while dolphins sampled in the summer showed stress-associated metabolic responses. Dolphins inhabiting areas previously reported to experience heavy vessel traffic and contaminant burdens exhibited enriched immune- and inflammation-associated pathways. Dolphins that visually appeared to have poorer body condition exhibited metabolite profiles suggestive of increased protein catabolism. Dolphins in extreme salinity conditions had more abundant membrane maintenance and endocrine pathways. **Conclusions**: Dolphins from each system exhibited distinct metabolic signatures that may be associated with differing physiological responses, highlighting the potential utility of blubber biomarkers for assessing physiological adaptations in free-ranging marine mammals. Improved understanding of habitat-specific physiological responses offers critical insights into how cumulative impacts may affect the health and adaptive capacity of vulnerable species in dynamic coastal ecosystems.

## 1. Introduction

Cetacean blubber is enriched in bioactive metabolites that regulate critical physiological processes, including lipid metabolism, hormone synthesis, electrolyte and energy balance, inflammation, and the acute-phase response [1,2,3,4,5]. The long lifespan, high trophic position, and lipid-rich blubber of cetaceans make them valuable sentinels for monitoring ecosystem-level environmental change [6,7,8]. Blubber thickness, lipid content, adipocyte size, and fatty acid composition are often used as proxies for energy storage and body condition to assess overall health status [9,10,11,12,13,14,15] but may not fully capture systemic biochemical shifts. Traditional targeted analytical approaches quantify only a relatively small number of metabolites simultaneously, limiting their ability to characterize broad physiological responses [16]. Due to the quantitative differences in metabolism between cetacean tissue types [17] and the long-term accumulation of biochemical changes in blubber relative to other tissue matrices [18], blubber metabolite profiles may provide insight into molecular adaptations to variable environmental conditions and better integrate longer-term physiological status in wild populations [5,19].

Metabolomics, the study of the entire metabolite composition of a tissue, is an emerging tool for monitoring the health and adaptations of free-ranging cetaceans to stressors [17,19,20]. Since the metabolome integrates an organism’s genetic, physiological, environmental, dietary, and microbial signals, systemic biochemical change(s) and physiological response(s) to stress can be monitored through observed shifts in metabolite composition or pathway regulation [20]. Untargeted metabolomic analyses enable the simultaneous assessment of a wide range of metabolites [21,22], providing comprehensive biomarker profiles and potentially advancing cetacean health monitoring in non-experimental settings. However, interpretation of untargeted metabolomic data may be constrained by challenges in metabolite identification and annotation, as many detected features cannot be confidently assigned to known compounds or biochemical pathways [23,24]. These limitations may be particularly pronounced in non-model species like cetaceans, for which species-specific metabolite databases and pathway annotations remain limited and often require broader mammalian reference databases for biological interpretation [25]. Accordingly, careful metabolite annotation and cautious biological interpretation are essential when applying untargeted metabolomics to wildlife species.

Despite these challenges, recent advances in cetacean omics-based approaches have successfully identified biomarkers associated with health status and environmental exposures in many small delphinids, such as the common bottlenose dolphin (*Tursiops truncatus*, hereafter ‘dolphin’). For instance, metabolomic analysis of breath condensates from dolphins in managed care facilities revealed biomarkers associated with cutaneous and respiratory infections, dental disease, and pregnancy [20]. Plasma metabolite profiles from free-ranging dolphins indicated reduced amino acid circulation in dolphins in poor health [11] and reduced sodium and chloride electrolytes in dolphins exposed to low-salinity conditions [26,27]. Untargeted metabolomic analyses of bottlenose dolphin blubber have been limited to stranded or post-mortem individuals and have identified metabolomic differences consistent with altered energy-metabolism pathways [17]. Although coastal dolphins can serve as effective bioindicators of ecosystem welfare [6,28,29], variation in the blubber metabolome of live dolphins inhabiting distinct coastal systems remains poorly understood. Metabolomic data are particularly valuable for dolphins in coastal bays, sounds, and estuaries (BSEs) influenced by shipping traffic, coastal development, and contaminant runoff or disposal, as these activities can alter estuarine nutrient regimes, disrupt foraging behaviors, and trigger systemic physiological changes [30,31,32]. Understanding how environmental variability and anthropogenic pressures are associated with metabolic variation in reproduction, immune response, and overall fitness is therefore warranted for coastal cetaceans [17,18,33].

Current cetacean models are largely focused on organism-level energetics (e.g., [34]), and recent studies that generated high-resolution biochemical data across multiple biological matrices [17,19,20] have not yet been incorporated into species-specific systems-level metabolic models. To our knowledge, this is the first study to perform untargeted blubber metabolomics in live, free-ranging bottlenose dolphins. We also develop a Systems Biology Markup Language (SBML) genome-scale metabolic model specifically for *Tursiops truncatus*, enabling species-specific pathway enrichment analysis and providing a framework for future flux balance simulations under varying environmental conditions. We characterized the blubber metabolomes of dolphins across three dynamic BSE systems along the South Texas coast (a protected seagrass nursery, an industrialized bay adjacent to a major international shipping port, and a hypersaline lagoon), each subject to distinct seasonal and anthropogenic pressures. We hypothesized that dolphins from these contrasting habitats would exhibit distinct blubber metabolomic signatures reflecting altered lipid, energy, and inflammatory metabolism.

## 2. Materials and Methods

### 2.1. Study Area and Stock Descriptions

**Figure 1:**
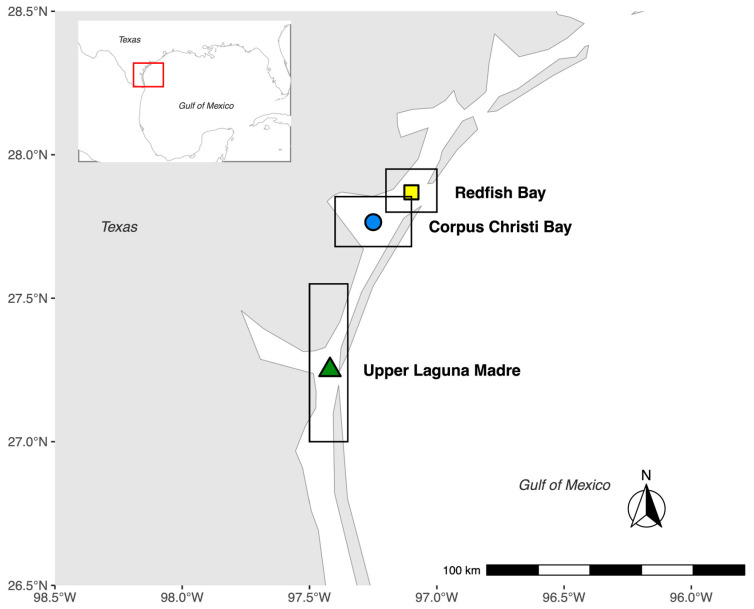
Map of field sampling locations consisting of Redfish Bay (yellow square), Corpus Christi Bay (blue circle), and Upper Laguna Madre (green triangle), Texas. Black rectangles around each site denote approximate sampling area. The red square in the inset map indicates the location of the study area within the broader geographic region. The map was generated in RStudio (v.2023.06.2+561).

The study area includes three adjacent BSE systems in South Texas: Redfish Bay (RB), Corpus Christi Bay (CCB), and Upper Laguna Madre (ULM) (Figure 1). Bottlenose dolphins in South Texas BSEs are considered highly vulnerable to climate change and are a high management priority by the National Oceanic and Atmospheric Administration (NOAA) due to habitat-specific environmental variability and numerous anthropogenic stressors [35,36,37]. RB is a protected seagrass nursery that supports commercially important fish [36], with primary production driven by large patches of turtle grass (*Thalassia testudinum*) and shoal grass (*Halodule wrightii*) [38]. Annual increases in average water temperature (22 °C) and salinity (26 ppt) [39] suggest rising thermal and osmotic pressures for biota in RB. Dolphin abundance in the channels surrounding RB coincides with seasonal fluctuation in water quality and prey availability, with dolphins exhibiting low site fidelity in the summer and high site fidelity in the winter [40]. Frequent dredging and commercial port activities in the channels surrounding RB subject the system to abundant vessel traffic, with vessels proximate to dolphins 80% of the time [41].

Adjacent to RB, CCB is a shallow, wind-driven estuary with the largest U.S. port in total revenue tonnage, >600 mineral production sites, and two superfund sites that have contributed to oil and heavy metal contamination of CCB and surrounding estuaries [36]. Primary production in CCB fluctuates with seasonal nutrient loads and water quality, with phytoplankton biomass peaking in spring-summer and declining in fall-winter in areas where seagrass vegetation is sparse [42]. Like RB, the average water temperature and salinity (26 °C and 32 ppt, respectively) have been increasing in CCB [39]. Dolphins exhibit high site fidelity in CCB during the winter, and large groups frequently follow shrimp trawlers near the shipping channel [40].

ULM extends directly southwest of CCB and is one of six hypersaline lagoons (>35 ppt) globally, with salinities regularly exceeding 45 ppt [43,44,45]. ULM is a highly productive ecosystem with primary production dominated by seagrass and microalgal communities [44]. As with RB and CCB, the average water temperature (24 °C) and salinity (40 ppt) in ULM are increasing annually [39]. Historical chemical and heavy metal pollution, together with emerging contaminants, have been documented in these systems and may represent environmental stressors for resident dolphins [36,46].

### 2.2. Data Collection

Blubber samples were collected from common bottlenose dolphins (*n* = 35) in the three South Texas BSEs (Table 1). During boat-based surveys in spring (May 2024) and summer (August 2024), blubber biopsies were obtained from live, free-ranging dolphins in RB, CCB (summer only), and ULM under federal and institutional permits (NMFS permit nos. 21938 and 27973; TAMU-CC-IACUC-2023-0011). Since dolphins from CCB were sampled only during summer, season and site-specific effects could not be fully separated in analyses involving this population. Blubber biopsies were collected below the dorsal fin (above the dolphin’s midline) using a crossbow (Barnett Blackcat, 74.8 kg draw weight; Barnett Outdoors, LLC, Tarpon Springs, FL, USA) and specialized darts (10 mm diameter tip; CETA-DART, Aarhus, Denmark) at a distance of 3–5 m from the animals [47]. Blubber samples were promptly retrieved, subsampled on ice into cryovials using a sterilized scalpel and petri dish, and stored in a dewar of liquid nitrogen until being returned to the lab. The average time between sample collection and transfer to liquid nitrogen was 17 min. Samples were stored at −80 °C in the lab within 12 h of collection. During all biopsy attempts, dolphins were photo-identified to ensure that individuals within and across seasons from each site were not resampled. Data were collected on salinity and water temperature (YSI Xylem Pro Solo, Yellow Springs, OH, USA), pH (YSI Ecosense pH 10A), group composition (number of dolphins; estimated age classes based on body size relative to the mother and mother/calf pairings, i.e., echelon position; predominant behavioral states for permit reporting), and biopsy sample metadata (number of tissue subsamples, location of dart contact with dolphin, individual and group reaction to biopsy, individual and group post-biopsy behavior). General body condition was noted opportunistically during field sampling based on observations of the visible body profile, but standardized quantitative body condition assessments were not conducted for dolphins included in the present study. Each group was monitored for strong behavioral responses to the biopsy dart deployment (e.g., breach, tail slap), and up to three attempts were made to biopsy any group. Biopsy attempts were terminated if new individuals joined the group, calves or young-of-year were present, or any individual in the group demonstrated strong behavioral responses. In this study, approximately 68% of dolphin groups were sampled once, 26% twice, and only 6% three times. Genetic sexing of the biopsied skin layer was performed *post hoc* using an established procedure [48]. Regions of the X and Y chromosomes were amplified by targeting the ZFX and SRY genes, respectively. Reproductive status of the sampled dolphins was not determined in this study. Potential effects of reproductive state on metabolite profiles could therefore not be evaluated.

### 2.3. Metabolite Extraction

Prior to metabolite extraction, blubber samples were stored at −80 °C for approximately one year. Metabolites were extracted from the blubber following an established protocol [47] with slight modifications. Approximately 50 mg of blubber tissue was minced on dry ice and homogenized in 1200 µL of 70:30 acetonitrile:water (LC-MS Optima grade, Fisher Scientific, Fair Lawn, NJ, USA). Samples were homogenized four times for 30 s at 6500 RPM with 5 min intervals on ice to maintain a stable temperature. Homogenized samples were centrifuged at 4 °C for 5 min at 7300 RPM and placed on a rotary shaker overnight at 4 °C and 7000 RPM. After at least 12 h, the supernatants were transferred into glass test tubes. An 800 µL aliquot of acetonitrile was added to the original homogenization tube, vortexed for 30 s, and centrifuged for 5 min at 4 °C and 7300 RPM to rinse the tubes. The supernatants were collected in glass tubes, and a 2.75 mL aliquot of hexane was added to remove fat from the acetonitrile layer. The samples were vortexed for 30 s and then placed on ice for 10 min to allow the layers to separate. The hexane layer (top layer) was extracted. The hexane rinse was repeated until no fat layer precipitated. An aliquot of 600 µL of lipid extracted sample was transferred to a centrifuge tube containing a 0.22 µm filter (Corning Costar Spin-X, Fort Worth, TX, USA) and centrifuged for 5 min at 4 °C and 7300 RPM. The procedure was repeated once more to filter the entire 1200 µL sample volume. The filtered samples were transferred to autosampler vials and stored at −20 °C until instrumental analysis.

### 2.4. Instrumental and Statistical Analysis

Blubber metabolites were analyzed in positive and negative ionization mode on a 1.7 μm ACQUITY UPLC BEH C18 reversed-phase column (Waters; 130 Å, 1.7 μm, 2.1 × 150 mm) on a Thermofisher Vanquish UHPLC system (Thermo Fisher Scientific, Germering, Germany) coupled with an Orbitrap Fusion mass spectrometer (Thermo Fisher Scientific, Bremen, Germany). As described in our previous work [49], internal standards were introduced post-column via T-shaped connections. In negative ionization mode, labeled hippuric acid (ring-^13^C_6_, 99%, Cambridge Isotope Laboratories) was used as the mass-locking standard, while labeled o-ketoisovaleric acid sodium salt (^13^C_5_, 98%, Cambridge Isotope Laboratories, Tewksbury, MA, USA) was used to monitor mass accuracy across the chromatographic run. In positive ionization mode, labeled proline (^13^C_5_, ^15^N, Sigma-Aldrich, MilliporeSigma, St. Louis, MO, USA) served as the mass-locking standard, while labeled valine (^13^C_5_, ^15^N, Sigma-Aldrich, MilliporeSigma, St. Louis, MO, USA) was used to assess mass accuracy throughout the retention time.

Untargeted instrumental analysis was achieved following an established protocol [47] with slight modifications. The total run time for reverse-phase chromatographic separation was set to 34 min, including a 7 min re-equilibration, with an injection volume of 20 µL. The heated electrospray ionization (H-ESI) settings were reduced to 2200 V for the positive spray and 2500 V for the negative spray, with the ion transfer tube temperature set to 325 °C and the vaporization temperature to 250 °C. The Orbitrap was operated at a 60,000 resolution (FWHM at *m*/*z* 200) over a mass range of 50–700 *m*/*z*, with the RF lens set to 40%. Pooled and blank (solvent only) samples were injected after every ten tissue samples as a quality control to compensate for ionization-induced signal drift [50]. To enhance MS/MS coverage and increase fragmentation depth, pooled samples were analyzed using the DeepScan ActiveX algorithm with four sequential injections. This iterative acquisition approach enabled increased MS/MS coverage of low-abundance and previously undersampled precursor ions.

Eleven samples representing the dataset’s spatial, temporal, and demographic variability were selected for a targeted exclusion analysis. Precursor ions that had not fragmented in prior runs were added to an exclusion list to prioritize fragmentation of previously uncharacterized compounds. The same instrumental parameters used in the untargeted analysis were maintained. Blubber UPLC-Orbitrap MS data were processed using Compound Discoverer v.3.5 (Thermo Fisher Scientific, Waltham, MA, USA) with a signal-to-noise ratio (S/N) ≥ 3 and a minimum of five scans per detected peak. All chromatography spectra retention times (RTs) were aligned using an adaptive curve with a maximum shift of 0.2 min and a 5 ppm mass tolerance. For structural elucidation, MS^2^ spectra were analyzed using the mzCloud database, an in-house structural database, and mzVault with a score threshold of ≥70%. *De novo* structural elucidation was based on MS^2^ fragment patterns, and putative annotations were generated using in silico fragmentation prediction software (Mass Frontier v.8.1). Fragment Ion Search (FISh) scoring was applied to compounds with both fragmentation and structural information, and annotations were verified using in silico fragmentation matching, with a FISh score threshold of ≥70%.

To minimize instrumental drift, inter-run variations, and systematic technical errors, data normalization was performed via Systematic Error Removal using the Random Forest (SERRF) algorithm. Pooled quality control (QC) samples, injected at 10-sample intervals throughout the analytical sequence, were utilized to monitor and control intra- and inter-day precision after applying SERRF. SERRF leveraged a random forest machine learning architecture to model and remove complex, intercorrelated systematic errors across sample runs, based on the variance observed in the QC injections. As shown in our previous works, this normalization process effectively reduced technical variation, minimizing the average technical error to a 5% relative standard deviation (RSD) and ensuring high reproducibility in sample analysis [49,51]. We used the post-alignment Fill Gaps algorithm in Compound Discoverer Software v.3.5 to address the missing values in some samples, with a mass tolerance of 5 ppm and an S/N threshold of 1.5. Metabolites with missing values in 25% or more of the samples were removed before further analysis.

Metabolite identification confidence was assigned according to the Metabolomics Standards Initiative (MSI) framework. Level 1 annotations were confirmed using authentic standards matching retention time, accurate mass, and MS/MS spectra. Level 2 annotations were based on high-confidence spectral matches to mzCloud or mzVault and/or strong FISh scores (≥70%) in the absence of standards. Level 3 annotations represented putative compound classes supported by diagnostic fragmentation or in silico prediction, while unmatched features were classified as Level 4 (unknowns). MSI levels were used to guide interpretation, with pathway analyses primarily based on Level 1–3 identifications.

Principal component analysis (PCA) of untargeted, normalized, mean-centered, and auto-scaled data from both negative and positive ionization modes was used to elucidate differences in blubber metabolomic profiles by dolphin sex, season, and sampling site. Estimated age class, reproductive status, and body condition were not included in statistical analyses, and no variables were statistically controlled. Since CCB dolphins were sampled only during summer, site-specific differences involving this population should be interpreted in the context of potential seasonal influences. Volcano plots were generated using univariate statistical testing (two-sample t-test by default) to visualize statistical significance (−log_10_ *p*-value) against fold change (log_2_). Metabolites were considered significantly different if *p* < 0.05 and |log_2_ fold change| ≥ 1. All PCA and volcano plot analyses were conducted in Compound Discoverer v.3.5. Given the unbalanced sampling design and limited subgroup sizes, multivariate models such as PERMANOVA or mixed-effects models were considered but not implemented because assumptions for robust inference could not be satisfied.

### 2.5. Systems Biology Markup Language (SBML) Model for Tursiops Truncatus

A genome-scale metabolic reconstruction for *Tursiops truncatus* was generated using an ortholog-based pipeline. The human genome-scale metabolic model (human-GEM) was used as the template network. Protein sequences for *T. truncatus* were retrieved in FASTA format from the NCBI RefSeq genome annotation (Annotation Release 101; assembly mTurTru1). These sequences were supplied to the CurveMe pipeline [52] for ortholog mapping and transferring gene–protein–reaction associations from the human template to the dolphin genome, resulting in a Systems Biology Markup Language (SBML) model specific for *T. truncatus*.

Building a *T. truncatus*-specific SBML-based genome-scale metabolic model offers a significant advantage over KEGG by shifting our analysis from isolated, generic genes to a stoichiometrically consistent, organism-specific network topology. While KEGG relies on static, human-defined pathway boundaries that treat pathways as disconnected lists, an SBML-GEM connects every reaction to its exact chemical substrates and products, allowing enrichment algorithms to trace continuous, unbroken subnetworks across traditional boundaries. Furthermore, because the SBML-GEM is manually curated for our *T. truncatus* species, it reduces the inclusion of biologically implausible reactions or dead-end pathways often found in automated KEGG maps and explicitly details cellular compartments and transport costs based on strict stoichiometry. This ensures that our enrichment results pinpoint true, biologically active metabolic crosstalk and functional shifts unique to *T. truncatus* species under that specific condition, rather than relying on statistical overlaps in a generalized reference catalog.

As the model is structured as an XML file, we utilized Python v.3.12.7 to extract the underlying network topology and map our experimental metabolomics data (including fold changes). Metabolic enrichment was then structurally computed by grouping associated reactions directly into functional subsystems. To integrate the metabolomics datasets, experimental mass-to-charge (*m*/*z*) features were matched to the theoretical monoisotopic masses of metabolites curated in the *T. truncatus* SBML-GEM. Matched metabolites were then filtered using log_2_fold-change thresholds to define subsets of significantly altered compounds. Rather than relying on rigid database definitions, functional pathway boundaries were derived directly from the metabolic network structure. Over-Representation Analysis (ORA) was then performed using a hypergeometric test [53,54] to compute metabolic enrichment by grouping associated reactions into functional subsystems. Finally, multiple testing correction was applied using the Benjamini–Hochberg procedure to control the false discovery rate (FDR).

Although the ortholog-based reconstruction pipeline using CurveMe and the Human-GEM template provided a rigorous framework for building the *T. truncatus* model, some limitations should be acknowledged. First, because the model was built using a template-driven approach, it may be biased toward human metabolism. As a result, specialized metabolic pathways unique to cetacean physiology or marine adaptation that lack clear human orthologs may not be represented in the current draft model. Second, while sequence-level orthology can indicate whether a reaction is present, it does not fully capture species-specific differences in regulation, tissue-specific expression, or enzyme kinetics that may be important for supporting the dolphin’s high-protein, high-fat, marine carnivorous diet. Finally, the model depends on the current *T. truncatus* RefSeq genome annotation; therefore, any uncharacterized, incomplete, or misannotated genes could lead to missing reactions or gaps in the metabolic network. Despite these limitations, this genome-scale model represents an important step beyond generic metabolic catalogs. It provides a computable framework that can be further refined as more cetacean-specific genomic, biochemical, and physiological data become available.

For metabolomics integration, experimental mass-to-charge (*m*/*z*) features were matched to the theoretical monoisotopic masses of metabolites in the *T. truncatus* SBML model. Matched metabolites were filtered based on log_2_ fold change thresholds to define significant subsets. Pathway enrichment analysis of all detected metabolites was performed using Over-Representation Analysis with a hypergeometric test [53,54], in which pathway definitions were derived directly from the metabolic network structure. Multiple testing correction was applied using the Benjamini–Hochberg procedure to control the false discovery rate (FDR; Table S1).

## 3. Results

**Figure 2:**
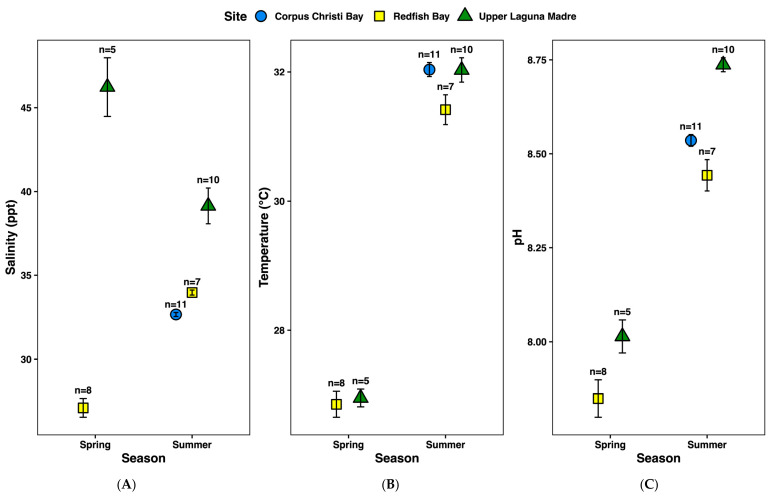
Water quality data ((**A**) salinity, (**B**) water temperature, and (**C**) pH) collected during biopsy surveys in spring and summer 2024. Water parameters are denoted as mean ± standard error for each site. Sites are denoted as Redfish Bay (yellow square), Corpus Christi Bay (blue circle), and Upper Laguna Madre (green triangle).

A total of 35 blubber samples were collected opportunistically from different dolphins between spring and summer 2024, including animals of different sex and age classes (all estimated to be juvenile or older) (Table 1). Salinity, water temperature, and pH differed descriptively among sampling locations and seasons (Figure 2); no formal statistical comparisons were performed because environmental measurements were intended to characterize sampling conditions rather than test ecological hypotheses. Collectively, 2777 metabolites (1002 in positive mode, 1775 in negative mode) were detected, of which *MS*^2^ fragmentation spectra were obtained for 1453 (544 in positive mode, 909 in negative mode); a list of confirmed (Level 1) and putatively annotated (Level 2) metabolites is presented in Table S2. Following SERRF normalization, the average relative standard deviation (RSD) of the peak areas for QC-detected metabolites was 6.7%. Because the same QC sample was injected at regular intervals throughout the run, this low RSD indicates excellent measurement reproducibility and minimal technical variation.

**Figure 3:**
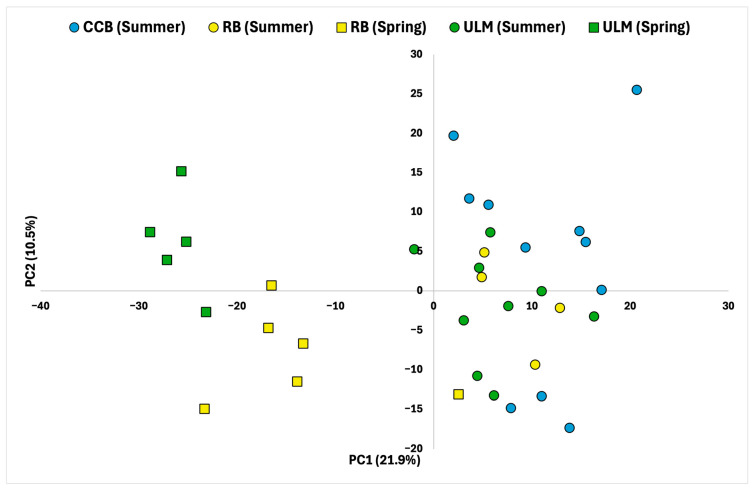
Principal component analysis (PCA) of the positive ionization mode untargeted metabolite profiles from common bottlenose dolphin (*Tursiops truncatus*) blubber across Redfish Bay (RB; yellow), Corpus Christi Bay (CCB; blue), and Upper Laguna Madre (ULM; green), Texas. Seasons are denoted by shape as spring (square) and summer (circle). PC1 reflects seasonal site-specific variation in metabolite composition, while PC2 distinguishes subsets of individuals within each site.

Principal component analysis (PCA) of the positive- and negative-ion-mode metabolomic data suggested partial visual separation of samples by season and site. The first two principal components of the positive-ion-mode data (Figure 3) explained 32.4% of the total variance (PC1 = 21.9%, PC2 = 10.5%) and should be interpreted with caution. Samples were primarily separated by season along PC1. Samples were separated by individuals within each system along PC2. The PCA of the negative ionization mode metabolomic data revealed a similar distribution, explaining 54.9% of the total variance (PC1 = 48.2%, PC2 = 6.7%) (Figure S1). PC1 primarily distinguished samples by season. However, all RB samples clustered together with the spring data. PC2 differentiated subsets of individuals within each site.

**Figure 4:**
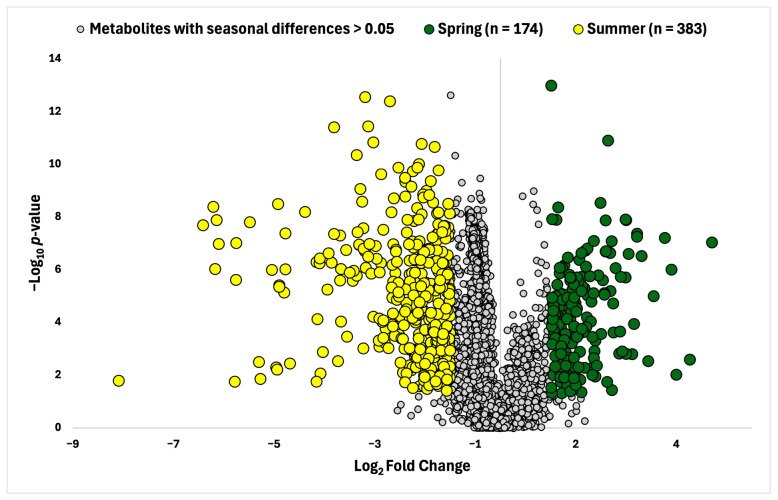
Volcano plot of all metabolites (circles) detected in positive and negative ionization mode. Metabolites with a positive Log_2_ value are considered to have higher abundance relative to those with a negative Log_2_ value (distinguished by the grey vertical line). Colored circles represent metabolites that are significantly different (*p* < 0.05) between spring (green) and summer (yellow), while grey circles represent metabolites that are not significantly different.

**Figure 5:**
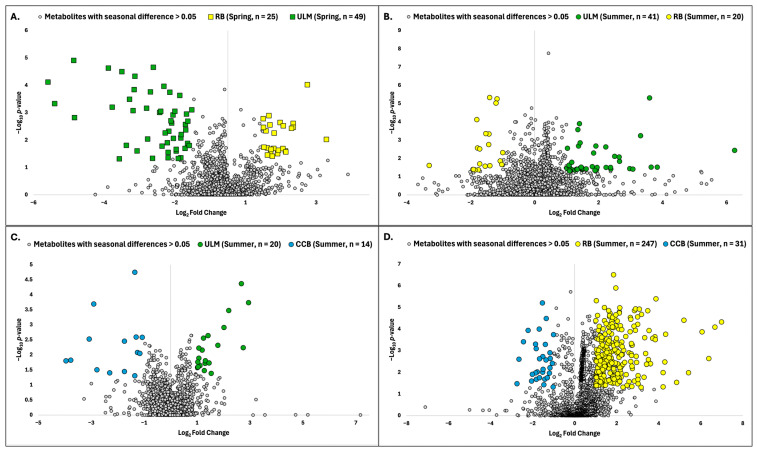
Volcano plots of all metabolites (circles) detected in positive and negative ionization modes, showing pairwise comparisons between sites and seasons: (**A**) RB versus ULM in spring, (**B**) RB versus ULM in summer, (**C**) CCB vs ULM in summer, (**D**) CCB versus RB in summer. Metabolites with a positive Log_2_ value are considered to have higher abundance relative to those with a negative Log_2_ value (distinguished by the grey vertical line). Colored circles represent metabolites that are significantly different (*p* > 0.05) between groups, while grey circles represent metabolites that are not significantly different. Sites are denoted by color (CCB = blue, RB = yellow, ULM = green) and season is denoted by shape (spring = box, summer = circle).

Volcano plot analyses of all detected metabolites in both positive and negative ionization modes identified significant seasonal patterns and site-specific differences in metabolite abundance (*p* < 0.05, |log_2_ fold change| ≥ 1) (Figure 4 and Figure 5). In total, 557 metabolites showed different abundances between spring and summer across all sites, with 174 metabolites showing significantly higher abundance (*p* < 0.05, |log_2_ fold change| ≥ 1) in spring and 383 metabolites showing lower abundance in summer (Figure 4). For example, citric acid (*p* = 1.9 × 10^−5^), tetradecanedioic acid (*p* = 2.5 × 10^−6^) and phenylacetylglycine (*p* = 0.01) were more abundant in spring, while lactic acid (*p* = 1.4 × 10^−9^), L-threonine (*p* = 1.9 × 10^−4^), and D-ribose (*p* = 4.2 × 10^−4^) were more abundant in summer.

**Figure 6:**
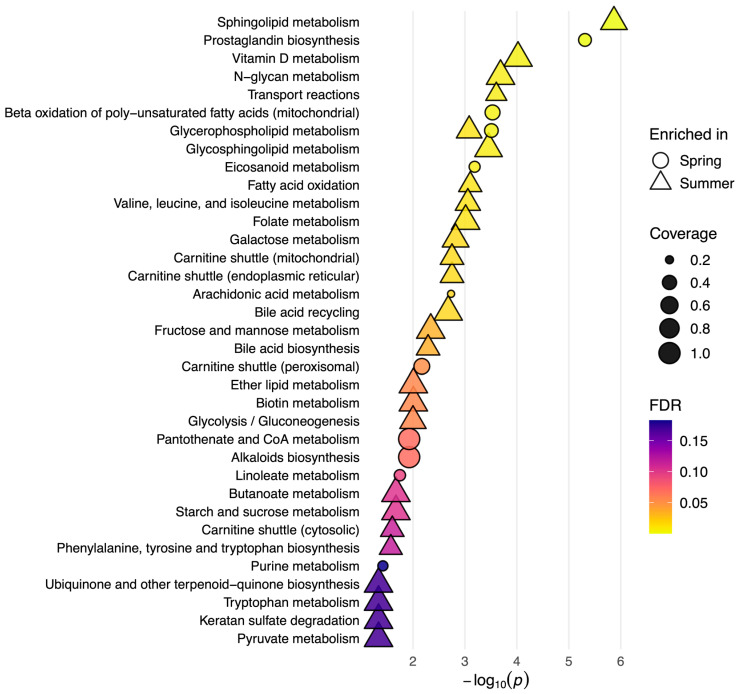
Pathway enrichment analysis showing pairwise comparisons of enriched metabolite pathways between spring and summer for all field sites combined. Pathways are denoted by color (FDR value), size (coverage), and shape (season enriched in).

Cumulative metabolite data revealed six major metabolite pathways, including lipid and energy metabolism, inflammatory and immune signaling, membrane integrity and cellular signaling, endocrine regulation, protein and amino acid turnover, and vitamin metabolism (Table 2). When metabolite data from the three sites were compared between spring and summer, 36 metabolic pathways were identified as significantly enriched (*p* < 0.05), indicating distinct seasonal regulation of lipid-derived metabolic processes (Figure 6, Table S1). Pathways enriched in spring were primarily related to fatty acid and eicosanoid metabolism, including prostaglandin biosynthesis, beta-oxidation of polyunsaturated fatty acids, arachidonic acid metabolism, and eicosanoid metabolism. Pathways enriched in summer were predominantly associated with lipid and energy metabolism, including sphingolipid metabolism, fatty acid oxidation, glycerophospholipid metabolism, and glycolysis/gluconeogenesis.

**Figure 7:**
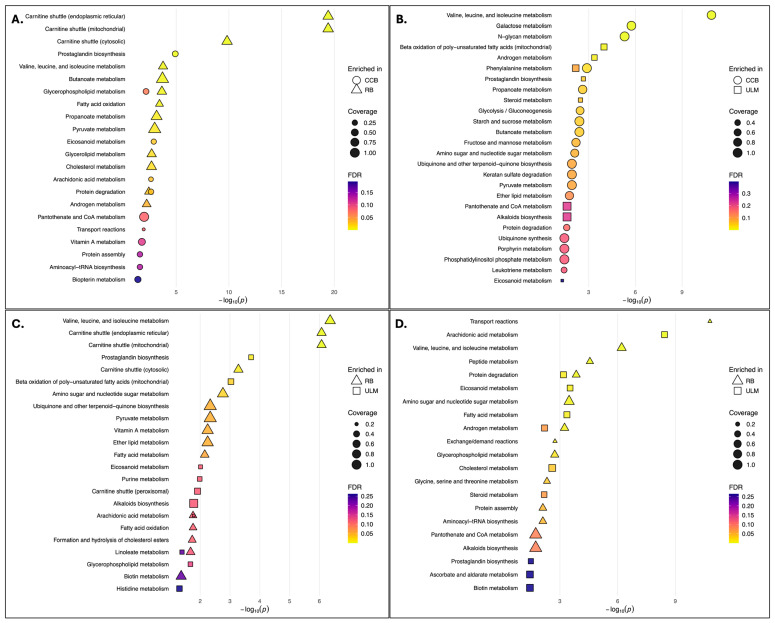
Pathway enrichment analysis showing pairwise comparisons of enriched metabolite pathways between field sites: (**A**) CCB versus RB in summer, (**B**) CCB versus ULM in summer, (**C**) RB vs ULM in summer, (**D**) RB versus ULM in spring. Pathways are denoted by color (FDR value), size (coverage), and shape (site enriched in).

Site-specific differences in metabolites occurred between and within seasons. Volcano plot analysis identified 557 metabolites and their associated pathways that were significantly different in abundance (*p* < 0.05, |log_2_ fold change| ≥ 1) across the three sites (Figure 7, Table 3).

## 4. Discussion

This is the first study to measure the metabolite profiles of blubber from live, free-ranging bottlenose dolphins. We demonstrate the utility of untargeted metabolomics as a powerful, minimally invasive, and complementary tool to direct assessments of seasonal physiological shifts in small cetaceans. The detected differences in blubber metabolite regulation are consistent with temporal and spatial variations in environmental conditions and human influences present at each site. We performed pathway enrichment using significantly altered metabolites mapped onto the SBML metabolic network (Table S1). We identified six major metabolite pathways: lipid and energy metabolism, inflammatory and immune signaling, membrane integrity and cellular signaling, endocrine regulation, and protein and amino acid turnover. These major pathways may reflect metabolic and physiological responses to environmental and anthropogenic stressors.

Recent advances in cetacean physiology have increasingly incorporated omics-based approaches to investigate endocrine function, metabolic regulation, contaminant exposure, and health status. Transcriptomic analyses have revealed extensive endocrine activity within dolphin blubber [5], while metabolomic studies of plasma and exhaled breath condensate have improved understanding of fasting physiology, disease progression, and cumulative responses to environmental stressors [20,32,55,56]. More recently, integrative multi-omics frameworks have provided systems-level insight into the biological processes underlying health and disease in odontocetes [19]. Together, these studies demonstrate the growing utility of molecular approaches for assessing cetacean physiology beyond traditional clinical biomarkers and morphometric assessments [8]. The present study extends the growing omics-based approaches by applying untargeted metabolomics to blubber from live, free-ranging dolphins, enabling evaluation of seasonal and spatial variation in metabolic pathways across environmentally distinct habitats.

### 4.1. Temporal Blubber Metabolite Patterns

Distinct seasonal differences in blubber metabolite profiles highlight how temporal environmental variability can shape the biochemical adaptations of dolphins. Samples collected in spring were characterized by enriched metabolite pathways associated with lipid quality (e.g., biologically active, energy-dense lipid pools), inflammatory signaling, and oxidative metabolism. Examples include beta-oxidation of polyunsaturated fatty acids, prostaglandin biosynthesis, eicosanoid metabolism, arachidonic acid metabolism, and linoleate metabolism. These pathways are closely linked to immune function [57], reproductive physiology [58], and basal inflammatory regulation [59,60]. This pattern may suggest a spring metabolic state oriented toward maintenance, regulation, coordination, and homeostasis rather than compensatory energy production. The concurrent enrichment of pantothenate and CoA metabolism and peroxisomal carnitine shuttle pathways in spring may reflect altered lipid metabolism and mitochondrial function when environmental conditions and prey resources are comparatively stable [61,62]. Because metabolite abundance does not directly measure pathway flux, these patterns could indicate either increased utilization of carnitine-mediated fatty acid transport or accumulation of biological intermediates.

Seasonal patterns in blubber metabolites become evident as summer approaches; there was a potential transition toward increased energy turnover, substrate flexibility, and broader macronutrient utilization. Pathways enriched in the summer were associated with lipid mobilization and transport, amino acid metabolism, and carbohydrate processing. These included sphingolipid and glycosphingolipid metabolism, fatty acid oxidation, the carnitine shuttle, BCAA metabolism, glycolysis/gluconeogenesis, and multiple sugar metabolism pathways. This pattern of enrichment may suggest a metabolically flexible state during late summer when elevated water temperatures, salinity, and seasonal shifts in prey availability may increase energetic costs [47,63,64]. Elevated fatty acid oxidation and carnitine shuttle pathways may further suggest an increased utilization of lipid reserves during summer, consistent with enhanced lipid metabolism in bottlenose dolphins during fasting and periods of elevated energetic demand [55,56]. Since we did not quantify dietary intake, reproductive state, and recent feeding history in this study, alternative explanations for the observed metabolomic differences cannot be excluded.

Commercially valuable spotted seatrout (*Cynoscion nebulosus*) and red drum (*Sciaenops ocellatus*) are common prey of dolphins in South Texas that exhibit habitat preferences based on seasonal salinity [65] and species resilience to extreme weather events [66]. Since prey abundance was not quantified in this study, interpretations of metabolic flexibility should be considered in the context of potential variation in prey availability across seasons. However, as measured values of salinity and water temperature increased consistently during the summer, shifts in ecosystem stability may reflect the enrichment of pathways needed to meet heightened energetic demands. Increased sphingolipid and glycerophospholipid metabolism may indicate active membrane remodeling and cellular signaling, processes consistent with membrane homeostasis and stress responses in mammals in response to thermal perturbations and other environmental stressors [67,68]. Elevated bile acid biosynthesis and recycling pathways may reflect altered lipid digestion and cholesterol turnover during periods of increased lipid mobilization [69]. Enriched butanoate metabolism may further support a reliance on alternative energy substrates and inflammatory regulation often observed under energetic or environmental stress [70,71,72].

### 4.2. Spatial Blubber Metabolite Patterns

Blubber metabolite pathways in RB dolphins were dominated by lipid mobilization, mitochondrial energy production, and endocrine and inflammatory regulation. Dolphins in RB repeatedly showed enrichment of carnitine shuttle pathways across mitochondrial, cytosolic, and endoplasmic reticular compartments. RB dolphins also exhibited increased fatty acid metabolism and oxidation, glycerolipid and glycerophospholipid metabolism, and cholesterol ester formation and hydrolysis. Enriched protein assembly and peptide metabolism may indicate tissue maintenance without progression toward extensive muscle catabolism [73]. Protected seagrass meadows within RB support abundant *C. nebulosus*, *S. ocellatus*, and black drum (*Pogonias cromis*) [36], providing relatively high-quality prey resources that are common to the diet of dolphins [74]. Seagrass habitats in RB are also influenced by surrounding mineral production and Superfund sites [36], which have the potential to introduce benthic pollutants into the food web [75]. Increased androgen metabolism in RB dolphins may reflect seasonal reproductive cycles [47]. Interactions with environmental stressors, including anthropogenic activity [36,76,77], should be considered. Previous studies have documented contaminant burdens in dolphins from these regions [46]; consequently, contaminant exposure represents one possible explanation for the observed metabolomic differences, although exposure was not measured in the present study.

RB dolphins were sampled in narrow channels with near-constant vessel traffic. Chronic vessel disturbance and acoustic exposure can increase metabolic expenditure in small odontocetes [78]. Enrichment of lipid mobilization and mitochondrial fatty acid oxidation pathways in RB dolphins may reflect sustained energetic demands associated with repeated vessel disturbance. Elevated eicosanoid and prostaglandin biosynthesis pathways may further indicate repeated anthropogenic disturbance, including chemical pollutant exposures [79], or heightened inflammatory signaling during seasonal reproductive cycles [80]. Although not directly measured in this study, interactions with environmental stressors may be a factor, given the documented presence of contaminants in regional dolphin tissues [45].

Despite the geographic proximity to RB, dolphins in CCB displayed distinct metabolite profiles associated with carbohydrate processing, protein turnover, glycolysis/gluconeogenesis, and multiple sugar metabolism pathways. We biopsied nine dolphins in CCB that were following shrimp trawl vessels and exhibited visual indicators of poor body condition (e.g., “peanut-head”, visible ribs) [81]. Since formal body condition metrics were not available for all sampled individuals, metabolomic patterns interpreted as reflecting energetic status could not be directly compared with quantitative measures of body condition. Additionally, metabolomic differences attributed to the site may partially reflect individual nutritional status. Trawler-associated dolphins showed enrichment of branched-chain amino acid (BCAA) metabolism and aminoacyl-tRNA biosynthesis, potentially suggesting an increased reliance on dietary amino acids for protein and energy synthesis. BCAAs are preferentially mobilized during periods of negative energy balance and have been linked to muscle catabolism in fasted dolphins [56] and fasted migrating humpback whales (*Megaptera novaeangliae*) [82]. This pattern is consistent with predictions for emaciated dolphins under nutritionally constrained conditions, where protein intake is episodic and prey have minimal energetic and nutritional value [56,70].

Aminoacyl-tRNA biosynthesis is also known to be highly responsive to cellular energy status and nutrient availability [55,83]. In model organisms such as mice, fasting and starvation induce coordinated suppression of global protein synthesis alongside reprogramming of tRNA charging and aminoacyl-tRNA synthetase activity [84,85]. In this context, enrichment of aminoacyl-tRNA biosynthesis pathways in trawler-associated dolphins may reflect metabolic stress and altered translational regulation associated with reduced or lower-quality prey intake. Odontocetes appear to lack functional ketogenic capacity [86], as demonstrated in experimental fasting studies in bottlenose dolphins [87] and comparative genomic evidence of reduced or non-functional hepatic ketogenesis [88]. In the absence of ketone body production, energy homeostasis during fasting is expected to rely more heavily on gluconeogenic mechanisms, which may utilize amino acids as glucose precursors when lipid stores become limited [9,82]. Concurrent elevation of protein degradation pathways may further indicate active protein turnover, consistent with reduced ketogenic capacity [32,62].

Trawler-associated dolphins in CCB showed increased butanoate metabolism, which regulates short-chain fatty acid-based energy production, lipid and cholesterol synthesis, and immune signaling [72]. This finding could further support a shift toward alternative energy substrates and inflammatory regulation. The butanoate metabolic pathway is sensitive to synthetic chemicals and heavy metals in marine turtles and delphinids [70,71,72], potentially affecting microbial function, fetal development, and systemic health and adaptation. Since the trawler-associated dolphins in CCB were sampled only in the summer, site-specific metabolomic differences in CCB cannot be fully disentangled from potential seasonal variation. The unbalanced seasonal sampling design precluded complete separation of site and seasonal effects in statistical comparisons involving CCB. Additional sampling of dolphins in CCB across seasons is warranted to distinguish spatial and temporal drivers of metabolomic variation and determine whether changes in prey availability may influence the metabolic patterns observed in this study.

In comparison with RB and CCB, dolphins inhabiting ULM exhibited blubber metabolite signatures consistent with sustained lipid utilization, membrane maintenance, and endocrine regulation under chronic hypersaline conditions. Repeated enrichment of fatty acid metabolism, beta-oxidation of polyunsaturated fatty acids, cholesterol metabolism, glycerophospholipid metabolism, and cholesterol ester formation suggest efficient lipid oxidation coupled with long-term lipid storage and membrane stability [89]. ULM is a shallow, hypersaline lagoon characterized by limited freshwater input, long water residence times, and comparatively low watershed-derived nutrient and contaminant loading [42,44]. Salinity and water temperature increase annually in ULM [39], potentially imposing additional osmotic and thermal pressures on resident biota already exposed to high salinities. Enrichment of prostaglandin biosynthesis, eicosanoid metabolism, arachidonic acid metabolism, and steroid metabolism may indicate coordinated inflammatory and endocrine signaling [90]; these processes are important for maintaining physiological homeostasis during environmental change. Since eicosanoids and steroid hormones regulate immune function, metabolism, reproduction, and cellular signaling, these pathways may reflect physiological adjustments to the osmotic and thermal variability characteristic of estuarine habitats [91].

Increased pantothenate and CoA metabolism, ascorbate and aldarate metabolism, and purine metabolism are consistent with altered cofactor availability, energy metabolism, and cellular redox regulation [92,93,94]. Although these pathways are associated with oxidative stress responses, they also play broader roles in energy metabolism, nucleotide turnover, immune function, and cellular homeostasis [92,93,94]. Their enrichment may therefore reflect physiological adjustments to the energetic and osmotic challenges of hypersaline environments [92,93,94]. Like RB, ULM is dominated by seagrass meadows that support abundant *C. nebulosus* and *P. cromis* [36], providing stable, high-quality prey resources compared to CCB. The dolphins sampled in ULM showed comparatively limited regulation of protein degradation pathways, further suggesting that energetic demands are met primarily through lipid-based metabolism rather than compensatory protein catabolism.

Finally, the cross-sectional design, relatively small sample size, and reliance on predominantly putative metabolite annotations limit the strength of causal inference. Future longitudinal studies incorporating repeated sampling, targeted metabolomics, contaminant analyses, and quantitative health assessments will be valuable for validating the hypotheses generated by this exploratory investigation.

## 5. Conclusions

Dolphin blubber metabolite profiles integrate signals of habitat quality, prey dynamics, seasonal variability, and anthropogenic influence. Geographically proximate systems may impose differing physiological pressures on resident dolphins. Blubber metabolite differences among the three sites ranged from efficient lipid-based energy metabolism and maintenance to coordinated shifts toward protein turnover and metabolic compensation, possibly in response to nutritional or environmental stress. Seasonal patterns were consistent with dynamic physiological responses; late-summer conditions may suggest energetic demands and metabolic flexibility, particularly in systems already characterized by anthropogenic disturbance and in individuals displaying visual indicators of poor body condition.

We present the first application of untargeted metabolomics to blubber samples collected from live, free-ranging bottlenose dolphins. Building on recent advances in cetacean physiology that increasingly incorporate omics-based approaches [17,19,55,81], we provide a novel, minimally invasive tool for investigating systemic metabolic regulation. Linking metabolite profiles to environmental context, prey availability, and known stressors advances marine mammal health research toward a mechanistic understanding of how dolphins physiologically and systemically respond to environmental variability and human impacts. By advancing metabolomic applications in non-model marine organisms, this study expands the use of biochemical profiling beyond traditional clinical and laboratory settings and provides a framework for integrating metabolomics into conservation physiology and environmental monitoring. Additionally, this study provides the first SBML model for *Tursiops truncatus*, improving metabolite identification and annotation in a non-model species and establishing a foundation for more accurate pathway-based analyses. This model provides a foundation for future simulations of species-specific flux balance analyses in response to environmental change. Expanding species-specific databases and incorporating long-term ecological and environmental data will improve interpretation of metabolomic responses and their relevance to conservation and management. Our findings underscore the value of metabolomics as an integrative tool for assessing sublethal stress, energetic balance, and resilience in dolphins, particularly in coastal systems undergoing rapid environmental change. As global anthropogenic pressures intensify, such approaches will be critical for identifying early physiological warning signs and informing conservation and management strategies to sustain healthy coastal dolphin stocks.

## Figures and Tables

**Table 1:** Biopsy sampling effort and demographics of blubber samples collected from common bottlenose dolphins (*Tursiops truncatus*) in 2024 in South Texas Bays, Sounds, and Estuaries (*n* = 35).

Site	Season Collected	*n*	Sex (Male:Female:Unknown)	Biopsy Attempts (% Success)	Adverse Reactions (%)
Redfish Bay	Spring Summer	6 4	1:3:2 2:1:1	28 (29%) 22 (32%)	3 (11%) 1 (5%)
Corpus Christi Bay	Summer	11	7:4:0	17 (59%)	2 (12%)
Upper Laguna Madre	Spring Summer	5 9	5:0:0 4:5:0	14 (36%) 19 (53%)	2 (14%) 2 (11%)

**Table 2:** Major metabolite pathways and the primary metabolites associated with each pathway that were detected in this study. Primary metabolites were detected in both positive and negative ionization modes.

Metabolite Pathway	Primary Metabolites Identified
Lipid and energy metabolism	Palmitic acid, acetyl-L-carnitine, propionylcarnitine, hexanoylcarnitine
Inflammatory and immune signaling	Taurine, 13-HODE
Membrane integrity and cellular signaling	Choline, dimethylsphingosine
Endocrine regulation	17α-Hydroxyprogesterone, norepinephrine
Protein and amino acid turnover	L-Phenylalanine, isoleucyl-valine, L-histidine (histidine derivatives), inosine
Vitamin metabolism	Nicotinamide

**Table 3:** Pairwise comparisons of blubber metabolites detected in dolphins (*n* = 35) from Redfish Bay, Corpus Christi Bay, and Upper Laguna Madre, Texas, during spring and summer. For each comparison, the numbers of significantly more and less abundant metabolites and enriched pathways are listed.

Comparison	Season	High-Abundance Metabolites	Low-Abundance Metabolites	Enriched Pathways
RB ^1^ vs. ULM ^2^	Spring	25 ^1^	49 ^2^	23
RB ^1^ vs. ULM ^2^	Summer	20 ^1^	41 ^2^	25
RB ^1^ vs. CCB ^2^	Summer	247 ^1^	31 ^2^	24
CCB ^1^ vs. ULM ^2^	Summer	14 ^1^	20 ^2^	27

^1^ Denotes metabolites associated with the first site listed in the corresponding comparison column. ^2^ Denotes metabolites associated with the second site listed in the corresponding comparison column.

## Data Availability

The raw UPLC-Orbitrap MS data (positive and negative mode) and Dolphin-SBML model presented in the study are openly available at Zenodo.org, DOI 10.5281/zenodo.20947629.

## Supplementary Materials

The following supporting information can be downloaded at: https://www.mdpi.com/article/10.3390/metabo16070473/s1, Table S1: Enriched metabolite pathways and their respective metabolic functions; Table S2: List of confirmed (Level 1) and putatively annotated (Level 2) metabolites; Figure S1: Principal component analysis (PCA) of the negative-ionization-mode untargeted metabolite profiles from common bottlenose dolphin (*Tursiops truncatus*) blubber; Figure S2: Principal component analysis (PCA) of the negative ionization mode untargeted metabolite profiles for common bottlenose dolphin (*Tursiops truncatus*) blubber during summer only; Figure S3: Heatmap of all detected metabolites in the positive ionization mode and their associations between seasons.

## Abbreviations

The following abbreviations are used in this manuscript:

BSE	Bay, sound, and estuary
GoM	Gulf of Mexico
RB	Redfish Bay
CCB	Corpus Christi Bay
ULM	Upper Laguna Madre
IAUCUC	Institutional Animal Care and Use Committee
NOAA	National Oceanic and Atmospheric Administration
NMFS	National Marine Fisheries Service
LC-MS	Liquid chromatography-mass spectrometry
RPM	Revolutions per minute
UHPLC	Ultra-high-performance liquid chromatography
FWHM	Full width at half maximum
RF	Radio frequency
RT	Retention time
MS/MS	Tandem mass spectrometry
S/N	Signal to noise
SERRF	Systematic error removal using random forest
QC	Quality control
MS^2^	Fragment scan
FISh	Fragment ion search
SBML	Systems biology markup language
FDR	False discovery rate
PCA	Principal component analysis
PC1	Principal component 1
PC2	Principal component 2
CoA	Coenzyme A
