# Supplementary material for: Untargeted Blubber Metabolomics Reveals Biochemical Signatures Associated with Physiological Status in Live, Free-Ranging Bottlenose Dolphins
Source: Metabolites. 2026 Jul 6;16(7):473. doi: 10.3390/metabo16070473 (PMC13413900; doi:10.3390/metabo16070473)
Supplement: Supplementary file 1 [file metabolites-16-00473-s001.zip › metabolites-4334620-supplementary.pdf]

Table S1. Enriched metabolite pathways and their respective metabolic functions. For each listed pathway, pathway size (n), the number of significant metabolites detected in the pathway, p-value, and false discovery rate (FDR) are reported.

Metabolite Pathway	Metabolic Function	Enriched in	Pathway Size (n)	Significant in Pathway (k)	P-Value	FDR
Sphingolipid metabolism	Synthesis and turnover of sphingolipids involved in membrane structure, cell signaling, and apoptosis	Summer	35	30	1E-06	0.0001
Vitamin D metabolism	Activation and regulation of vitamin D for calcium balance and immune modulation		12	12	1E-04	0.0047
N-glycan metabolism	Processing of N-linked glycans critical for protein folding, stability, and cell signaling		11	11	2E-04	0.0061
Transport reactions	Movement of metabolites across cellular membranes to maintain metabolic homeostasis		1362	672	2E-04	0.0061
Glycosphingolipid metabolism	Production and degradation of glycosphingolipids involved in membrane integrity and cell recognition		14	13	4E-04	0.0069
Fatty acid oxidation	Breakdown of fatty acids to generate		107	66	8E-04	0.0106

	ATP through β-oxidation					
Glycerophospholipid metabolism	Synthesis and remodeling of membrane phospholipids and signaling lipids		27	21	8E-04	0.0106
Valine, leucine, and isoleucine metabolism	Catabolism of branched-chain amino acids for energy production and metabolic regulation		32	24	9E-04	0.0106
Folate metabolism	One-carbon transfer reactions essential for DNA synthesis and methylation		9	9	1E-03	0.0106
Galactose metabolism	Conversion of galactose into glucose intermediates for energy production		15	13	2E-03	0.0145
Carnitine shuttle (endoplasmic reticular)	Transport of fatty acids for lipid remodeling and metabolic processing		72	46	2E-03	0.0145
Carnitine shuttle (mitochondrial)	Transport of long-chain fatty acids into mitochondria for β-oxidation		72	46	2E-03	0.0145
Bile acid recycling	Reabsorption and reuse of bile acids to maintain lipid digestion efficiency		8	8	2E-03	0.0158
Fructose and mannose metabolism	Conversion of hexose sugars into glycolytic intermediates		7	7	5E-03	0.0318
Bile acid biosynthesis	Conversion of cholesterol into		58	37	5E-03	0.0334

	bile acids for lipid digestion and cholesterol regulation					
Biotin metabolism	Regulation of biotin-dependent carboxylation reactions in fatty acid and glucose metabolism		6	6	1E-02	0.0544
Ether lipid metabolism	Synthesis of ether-linked lipids important for membrane stability and oxidative protection		6	6	1E-02	0.0544
Glycolysis / Gluconeogenesis	Breakdown of glucose for ATP production and synthesis of glucose from non-carbohydrate precursors		12	10	1E-02	0.0544
Starch and sucrose metabolism	Conversion of complex carbohydrates into metabolically usable sugars		5	5	2E-02	0.1043
Butanoate metabolism	Metabolism of short-chain fatty acids for energy and signaling functions		5	5	2E-02	0.1043
Carnitine shuttle (cytosolic)	Activation and preparation of fatty acids for intracellular transport and oxidation		36	23	3E-02	0.1167
Phenylalanine, tyrosine and	Production of aromatic amino acids involved in		53	32	3E-02	0.1189

tryptophan biosynthesis	protein synthesis and neurotransmitter precursors					
Pyruvate metabolism	Central metabolic node linking glycolysis, the TCA cycle, and amino acid metabolism		4	4	5E-02	0.1735
Keratan sulfate degradation	Breakdown of glycosaminoglycans involved in extracellular matrix remodeling		4	4	5E-02	0.1735
Tryptophan metabolism	Conversion of tryptophan into serotonin, kynurenine, and other bioactive metabolites		4	4	5E-02	0.1735
Ubiquinone and other terpenoid-quinone biosynthesis	Production of electron transport chain components essential for oxidative phosphorylation		4	4	5E-02	0.1735
Prostaglandin biosynthesis	Formation of lipid mediators involved in inflammation and vascular regulation	Spring	58	19	5E-06	0.0003
Beta oxidation of poly-unsaturated fatty acids (mitochondrial)	Energy production from long-chain polyunsaturated fatty acids		18	8	3E-04	0.0041
Glycerophospholipid metabolism	Remodeling of phospholipids for membrane		27	10	3E-04	0.0041

	function and signaling					
Eicosanoid metabolism	Synthesis of bioactive lipid mediators that regulate inflammation and immunity		62	16	7E-04	0.0069
Arachidonic acid metabolism	Conversion of arachidonic acid into inflammatory and signaling molecules		127	25	2E-03	0.0163
Carnitine shuttle (peroxisomal)	Transport of very-long-chain fatty acids into peroxisomes for oxidation		8	4	7E-03	0.0512
Alkaloids biosynthesis	Production of nitrogen-containing secondary metabolites involved in defense and signaling		2	2	1E-02	0.0697
Pantothenate and CoA metabolism	Synthesis of coenzyme A for fatty acid metabolism and the TCA cycle		2	2	1E-02	0.0697
Linoleate metabolism	Conversion of linoleic acid into bioactive lipid mediators		26	7	2E-02	0.0947
Purine metabolism	Synthesis and degradation of purine nucleotides for DNA, RNA, and energy transfer (ATP, GTP)		30	7	4E-02	0.1828

Table S2. List of confirmed (Level 1) and putatively annotated (Level 2) metabolites.

**Metabolitie**	**Formula**	**Confirmed Identification Level**
Urea	C H4 N2 O	Level 1: Confirmed Identification
Inosine	C10 H12 N4 O5	Level 1: Confirmed Identification
Adenosine	C10 H13 N5 O4	Level 1: Confirmed Identification
Propionylcarnitine	C10 H19 N O4	Level 1: Confirmed Identification
Decanamide	C10 H21 N O	Level 1: Confirmed Identification
Triethylene glycol monobutyl ether	C10 H22 O4	Level 1: Confirmed Identification
DL-Tryptophan	C11 H12 N2 O2	Level 1: Confirmed Identification
(5E,7E)-3-aminoundeca-5,7-dienoic acid	C11 H19 N O2	Level 1: Confirmed Identification
Leu-Pro	C11 H20 N2 O3	Level 1: Confirmed Identification
trans-3-Indoleacrylic acid	C11 H9 N O2	Level 1: Confirmed Identification
4-oxo-4-[(1-phenylethyl)amino]but-2-enoic acid	C12 H13 N O3	Level 1: Confirmed Identification
alpha-Hydroxy-1-methyl-1H-indole-3-propanoic acid	C12 H13 N O3	Level 1: Confirmed Identification
Phlorin	C12 H16 O8	Level 1: Confirmed Identification
1,4-Dimethyl-5H-pyrido[4,3-b]indol-3-amine	C13 H13 N3	Level 1: Confirmed Identification
Hexanoylcarnitine	C13 H25 N O4	Level 1: Confirmed Identification
Gly-Met-Arg	C13 H26 N6 O4 S	Level 1: Confirmed Identification
Rishitin	C14 H22 O2	Level 1: Confirmed Identification
4-Oxo-4-[(3-oxo-2-decanyl)amino]butanoic acid	C14 H25 N O4	Level 1: Confirmed Identification
Valerenic acid	C15 H22 O2	Level 1: Confirmed Identification
Di-n-butyl phthalate	C16 H22 O4	Level 1: Confirmed Identification
1,4-Anhydro-5-[(cyclopentylcarbonyl)amino]-2,5-dideoxy-2-[(2,2-dimethylpropyl)amino]-D-arabinitol	C16 H30 N2 O3	Level 1: Confirmed Identification
Palmitic acid	C16 H32 O2	Level 1: Confirmed Identification
8-Hydroxyhexadecanoic acid	C16 H32 O3	Level 1: Confirmed Identification
Hexadecanamide	C16 H33 N O	Level 1: Confirmed Identification
8-{3-Oxo-2-[(2E)-2-penten-1-yl]-1-cyclopenten-1-yl}octanoic acid	C18 H28 O3	Level 1: Confirmed Identification
Linolenic acid	C18 H30 O2	Level 1: Confirmed Identification
2-Aminooctadec-4-yne-1,3-diol	C18 H35 N O2	Level 1: Confirmed Identification

D-Sphingosine	$C_{18}H_{37}NO_2$	Level 1: Confirmed Identification
Tris(2-butoxyethyl) phosphate	$C_{18}H_{39}O_7P$	Level 1: Confirmed Identification
1-[4-(2-Hydroxyethyl)-1-piperazinyl]-2-[(1S,4S,5S)-4-(hydroxymethyl)-5-isopropyl-2-methyl-2-cyclohexen-1-yl]ethanone	$C_{19}H_{34}N_2O_3$	Level 1: Confirmed Identification
16-(2,3-Dihydroxypropoxy)-16-oxohexadecanoic acid	$C_{19}H_{36}O_6$	Level 1: Confirmed Identification
Gln-Tyr-Ile	$C_{20}H_{30}N_4O_6$	Level 1: Confirmed Identification
(±)8(9)-EpETE	$C_{20}H_{30}O_3$	Level 1: Confirmed Identification
Poly THF n5	$C_{20}H_{42}O_6$	Level 1: Confirmed Identification
Tetrahydrocurcumin	$C_{21}H_{24}O_6$	Level 1: Confirmed Identification
2,5-Anhydro-4,6-dideoxy-6-{[(1-methyl-1H-pyrazol-4-yl)carbonyl]amino}-4-(4-phenyl-1-piperazinyl)-D-galactitol	$C_{21}H_{29}N_5O_4$	Level 1: Confirmed Identification
17-alpha-Hydroxyprogesterone	$C_{21}H_{30}O_3$	Level 1: Confirmed Identification
methyl (1R,2R,3S,4S,5R,9S,13R,14S)-2,3,14-trihydroxy-14-(hydroxymethyl)-5,9-dimethyltetracyclo[11.2.1.01,10.04,9]hexadecane-5-carboxylate	$C_{21}H_{34}O_6$	Level 1: Confirmed Identification
Bis(methylbenzylidene)sorbitol	$C_{22}H_{26}O_6$	Level 1: Confirmed Identification
Erucamide	$C_{22}H_{43}NO$	Level 1: Confirmed Identification
(1R,9S)-11-(2-Methoxybenzoyl)-3-(5-pyrimidinyl)-7,11-diazatricyclo[7.3.1.02,7]trideca-2,4-dien-6-one	$C_{23}H_{22}N_4O_3$	Level 1: Confirmed Identification
Palmitoylcarnitine	$C_{23}H_{45}NO_4$	Level 1: Confirmed Identification
Bis(4-ethylbenzylidene)sorbitol	$C_{24}H_{30}O_6$	Level 1: Confirmed Identification
1-{[(1S,4S,6S)-6-Isopropyl-3-methyl-4-{[5-(1-methyl-1H-imidazol-5-yl)-1,3,4-oxadiazol-2-yl]methyl}-2-cyclohexen-1-yl]methyl}-4-piperidinecarboxamide	$C_{24}H_{36}N_6O_2$	Level 1: Confirmed Identification

2-[(1S,4S,5S)-4-{[(Cyclohexylcarbamoyl)amino]methyl}-5-isopropyl-2-methyl-2-cyclohexen-1-yl]-N-[2-(dimethylamino)ethyl]acetamide	C24 H44 N4 O2	Level 1: Confirmed Identification
1-Palmitoyl-sn-glycero-3-Phosphocholine	C24 H50 N O7 P	Level 1: Confirmed Identification
4-(Dimethylamino)-N-{[(2R,4S,5S)-5-{[4-(2-pyrimidinyl)-1-piperazinyl]methyl}-1-azabicyclo[2.2.2]oct-2-yl]methyl}benzamide	C26 H37 N7 O	Level 1: Confirmed Identification
2-[(1S,4S,5S)-4-{[(Cyclohexylcarbamoyl)amino]methyl}-5-isopropyl-2-methyl-2-cyclohexen-1-yl]-N-(3-pyridinylmethyl)acetamide	C26 H40 N4 O2	Level 1: Confirmed Identification
Taurocholic acid	C26 H45 N O7 S	Level 1: Confirmed Identification
Linoleic acid-biotin	C28 H48 N4 O3 S	Level 1: Confirmed Identification
Creatinine	C4 H7 N3 O	Level 1: Confirmed Identification
5-Aminopentanoic acid	C5 H11 N O2	Level 1: Confirmed Identification
L-methionine	C5 H11 N O2 S	Level 1: Confirmed Identification
Choline	C5 H13 N O	Level 1: Confirmed Identification
2-Hydroxypurine	C5 H4 N4 O	Level 1: Confirmed Identification
2-Piperidinone	C5 H9 N O	Level 1: Confirmed Identification
L-Proline	C5 H9 N O2	Level 1: Confirmed Identification
L-Pipecolinic acid	C6 H11 N O2	Level 1: Confirmed Identification
D-(-)-Tagatose	C6 H12 O6	Level 1: Confirmed Identification
L-isoleucine	C6 H13 N O2	Level 1: Confirmed Identification
L-Norleucine	C6 H13 N O2	Level 1: Confirmed Identification
Nicotinamide	C6 H6 N2 O	Level 1: Confirmed Identification
N1-(2-amino-2-oxoethyl)-2-(isopropylthio)acetamide	C7 H14 N2 O2 S	Level 1: Confirmed Identification
Acetylcholine	C7 H15 N O2	Level 1: Confirmed Identification
N-(2-Hydroxyethyl)valine	C7 H15 N O3	Level 1: Confirmed Identification
2,4-Dimethylphenol	C8 H10 O	Level 1: Confirmed Identification
DL-PHENYLALANINE	C9 H11 N O2	Level 1: Confirmed Identification
Gentiatibetine	C9 H11 N O2	Level 1: Confirmed Identification
L-Tyrosine	C9 H11 N O3	Level 1: Confirmed Identification

Acetyl-L-carnitine	C9 H17 N O4	Level 1: Confirmed Identification
2,2,6,6-Tetramethyl-1-piperidinol (TEMPO)	C9 H19 N O	Level 1: Confirmed Identification
Methylenediphosphonic acid	C H6 O6 P2	Level 2: Putatively Annotated Compounds
4-(Methylnitrosamino)-1-(3-pyridyl)-1-butanol	C10 H15 N3 O2	Level 2: Putatively Annotated Compounds
Prolylproline	C10 H16 N2 O3	Level 2: Putatively Annotated Compounds
Homocarnosine	C10 H16 N4 O3	Level 2: Putatively Annotated Compounds
Dethiobiotin	C10 H18 N2 O3	Level 2: Putatively Annotated Compounds
Valylvaline	C10 H20 N2 O3	Level 2: Putatively Annotated Compounds
L-decyline	C10 H21 N O2	Level 2: Putatively Annotated Compounds
(2S,8R)-2-Amino-8-hydroxydecanoic acid	C10 H21 N O3	Level 2: Putatively Annotated Compounds
6-Methylquinoline	C10 H9 N	Level 2: Putatively Annotated Compounds
1,2,3,4-Tetrahydro-beta-carboline	C11 H12 N2	Level 2: Putatively Annotated Compounds
1-(4-Methyl-5-thiazolyl)-1-phenylmethylamine	C11 H12 N2 S	Level 2: Putatively Annotated Compounds
N-Acetyl-L-phenylalanine	C11 H13 N O3	Level 2: Putatively Annotated Compounds
(R)-1,2-dimethyl-5,6-dihydroxy-tetrahydroisoquinoline	C11 H15 N O2	Level 2: Putatively Annotated Compounds
Jasmolone	C11 H16 O2	Level 2: Putatively Annotated Compounds
BAPRTEOLNVFKBX-UHFFFAOYSA-N	C11 H17 F7 O	Level 2: Putatively Annotated Compounds
gamma-Glutamylisoleucine	C11 H20 N2 O5	Level 2: Putatively Annotated Compounds
Undecylenic acid	C11 H20 O2	Level 2: Putatively Annotated Compounds
Butyrylcarnitine	C11 H21 N O4	Level 2: Putatively Annotated Compounds
Isoleucyl-Valine	C11 H22 N2 O3	Level 2: Putatively Annotated Compounds

Arginylglutamine	C11 H22 N6 O4	Level 2: Putatively Annotated Compounds
11-Aminoundecanoic acid	C11 H23 N O2	Level 2: Putatively Annotated Compounds
Methionyl-Lysine	C11 H23 N3 O3 S	Level 2: Putatively Annotated Compounds
1-Methyl-4-phenyl-1,2,3,6-tetrahydropyridine	C12 H15 N	Level 2: Putatively Annotated Compounds
5,5-Dimethyl-2-phenylmorpholine	C12 H17 N O	Level 2: Putatively Annotated Compounds
N-Cyano-N'-(1,1-dimethylpropyl)-N''-(3-pyridinyl)guanidine	C12 H17 N5	Level 2: Putatively Annotated Compounds
Hexylresorcinol	C12 H18 O2	Level 2: Putatively Annotated Compounds
(8aS)-7-(Tetrahydro-2H-pyran-4-ylcarbonyl)hexahydroimidazo[1,5-a]pyrazin-3(2H)-one	C12 H19 N3 O3	Level 2: Putatively Annotated Compounds
Traumatic acid	C12 H20 O4	Level 2: Putatively Annotated Compounds
N-OCTANOYL-L-HOMOSERINE LACTONE	C12 H21 N O3	Level 2: Putatively Annotated Compounds
2-Methylbutyroylcarnitine	C12 H23 N O4	Level 2: Putatively Annotated Compounds
3-Hydroxyvalerylcarnitine	C12 H23 N O5	Level 2: Putatively Annotated Compounds
hydroxyisovaleroyl carnitine	C12 H23 N O5	Level 2: Putatively Annotated Compounds
Isoleucyl-Isoleucine	C12 H24 N2 O3	Level 2: Putatively Annotated Compounds
Dodecanamide	C12 H25 N O	Level 2: Putatively Annotated Compounds
(7E)-3,8-Dimethyl-7-decen-1-yl trihydrogen diphosphate	C12 H26 O7 P2	Level 2: Putatively Annotated Compounds
Fluorene	C13 H10	Level 2: Putatively Annotated Compounds
N-Acetyltryptophan	C13 H14 N2 O3	Level 2: Putatively Annotated Compounds
Dioscorine	C13 H19 N O2	Level 2: Putatively Annotated Compounds
2-Carboxy-4-dodecanolide	C13 H22 O4	Level 2: Putatively Annotated Compounds

Hexenoylcarnitine	C13 H23 N O4	Level 2: Putatively Annotated Compounds
Leucylleucine methyl ester	C13 H26 N2 O3	Level 2: Putatively Annotated Compounds
2',3-Dimethyl-4-aminobiphenyl	C14 H15 N	Level 2: Putatively Annotated Compounds
7,8-dimethyl-1,2,3,4-tetrahydrophenazine	C14 H16 N2	Level 2: Putatively Annotated Compounds
Phenylalanylproline	C14 H18 N2 O3	Level 2: Putatively Annotated Compounds
L-Menthyl acetoacetate	C14 H24 O3	Level 2: Putatively Annotated Compounds
Hept-3-enoylcarnitine	C14 H25 N O4	Level 2: Putatively Annotated Compounds
Cellulose, microcrystalline	C14 H26 O11	Level 2: Putatively Annotated Compounds
5-Methylhexanoylcarnitine	C14 H27 N O4	Level 2: Putatively Annotated Compounds
Heptanoylcarnitine	C14 H27 N O4	Level 2: Putatively Annotated Compounds
n-octyl-beta-D-thioglucopyranoside	C14 H28 O5 S	Level 2: Putatively Annotated Compounds
Myristamide	C14 H29 N O	Level 2: Putatively Annotated Compounds
Heptaethylene glycol	C14 H30 O8	Level 2: Putatively Annotated Compounds
Xestoaminol C	C14 H31 N O	Level 2: Putatively Annotated Compounds
1-Tert-butyl-3-(4-chlorophenyl)-1H-pyrazolo[3,4-d]pyrimidin-4-amine	C15 H16 Cl N5	Level 2: Putatively Annotated Compounds
(S)-Pterosin A	C15 H20 O3	Level 2: Putatively Annotated Compounds
Leucylphenylalanine	C15 H22 N2 O3	Level 2: Putatively Annotated Compounds
Rotundine B	C15 H23 N O	Level 2: Putatively Annotated Compounds
L-Prolinamide, 5-oxo-L-prolyl-L-norvalyl-	C15 H24 N4 O4	Level 2: Putatively Annotated Compounds
2-(4-Hydroxy-4,8-dimethyl-6-oxo-7-propan-2-yl-1-bicyclo[3.2.1]octanyl)acetic acid	C15 H24 O4	Level 2: Putatively Annotated Compounds

4,11,13,15-Tetrahydroridentin B	C15 H24 O4	Level 2: Putatively Annotated Compounds
(1R,7aS)-1-[[[(2S,3R)-2,3-Dihydroxy-2-isopropylbutanoyl]oxy]methyl]hexahydro-1H-pyrrolizine 4-oxide	C15 H27 N O5	Level 2: Putatively Annotated Compounds
3-Hydroxy-cis-5-octenoylcarnitine	C15 H27 N O5	Level 2: Putatively Annotated Compounds
1-Dodecylimidazole	C15 H28 N2	Level 2: Putatively Annotated Compounds
Dihydro-2,4,6-tris(2-methylpropyl)-4h-1,3,5-dithiazine	C15 H31 N S2	Level 2: Putatively Annotated Compounds
Prolyl-Tryptophan	C16 H19 N3 O3	Level 2: Putatively Annotated Compounds
Hydroxyprolyl-Tryptophan	C16 H19 N3 O4	Level 2: Putatively Annotated Compounds
Dimethylbenzyl carbinyl hexanoate	C16 H24 O2	Level 2: Putatively Annotated Compounds
3-Methyl-alpha-ionyl acetate	C16 H26 O2	Level 2: Putatively Annotated Compounds
4,7,10,13-Hexadecatetraenoic acid	C16 H26 O2	Level 2: Putatively Annotated Compounds
Norambreinolide	C16 H26 O2	Level 2: Putatively Annotated Compounds
4-Hydroxy-3-methoxy-2,10-bisaboladien-9-one	C16 H26 O3	Level 2: Putatively Annotated Compounds
Nona-2,5-dienoylcarnitine	C16 H27 N O4	Level 2: Putatively Annotated Compounds
Zingiberol	C16 H28 O	Level 2: Putatively Annotated Compounds
1-(3-Methylbutanoyl)-6-apiosylglucose	C16 H28 O11	Level 2: Putatively Annotated Compounds
Tetranorprostanedioic acid	C16 H28 O4	Level 2: Putatively Annotated Compounds
6-Hydroxynon-7-enoylcarnitine	C16 H29 N O5	Level 2: Putatively Annotated Compounds
pre-putrebactin	C16 H30 N4 O7	Level 2: Putatively Annotated Compounds
Palmitoleamide	C16 H31 N O	Level 2: Putatively Annotated Compounds

Octyl (4ξ)-2-deoxy-2-[(Z)-(1-hydroxyethylidene)amino]-α-D-xylo-hexopyranoside	C16 H31 N O6	Level 2: Putatively Annotated Compounds
Hexadecasphingosine	C16 H33 N O2	Level 2: Putatively Annotated Compounds
Isoeugenol benzyl ether	C17 H18 O2	Level 2: Putatively Annotated Compounds
3-Methoxymorphinan	C17 H23 N O	Level 2: Putatively Annotated Compounds
Shogaol	C17 H24 O3	Level 2: Putatively Annotated Compounds
ACRL Toxin II	C17 H24 O5	Level 2: Putatively Annotated Compounds
(8aR)-2-Cyclohexyl-7-[(1-methyl-1H-imidazol-2-yl)methyl]hexahydroimidazo[1,5-a]pyrazin-3(2H)-one	C17 H27 N5 O	Level 2: Putatively Annotated Compounds
Tanacetol B	C17 H28 O4	Level 2: Putatively Annotated Compounds
(6E)-8-Methylnon-6-enoylcarnitine	C17 H31 N O4	Level 2: Putatively Annotated Compounds
5-Hydroxydec-5-enoylcarnitine	C17 H31 N O5	Level 2: Putatively Annotated Compounds
Sebacoyl-L-carnitine	C17 H31 N O6	Level 2: Putatively Annotated Compounds
N-Lauroyl Valine	C17 H33 N O3	Level 2: Putatively Annotated Compounds
3,8-Dihydroxydecanoylcarnitine	C17 H33 N O6	Level 2: Putatively Annotated Compounds
1-(9Z-tetradecenoyl)-glycero-3-phosphate	C17 H33 O7 P	Level 2: Putatively Annotated Compounds
Indolocarbazole	C18 H10 N2	Level 2: Putatively Annotated Compounds
2-Hydroxy-1-naphthaldehyde salicyloylhydrazone	C18 H14 N2 O3	Level 2: Putatively Annotated Compounds
Agestricin A	C18 H16 O7	Level 2: Putatively Annotated Compounds
2-Diphenylmethylpiperidine	C18 H21 N	Level 2: Putatively Annotated Compounds
Estriol	C18 H24 O3	Level 2: Putatively Annotated Compounds

19-Norandrosterone	C18 H28 O2	Level 2: Putatively Annotated Compounds
Methylgingerol	C18 H28 O4	Level 2: Putatively Annotated Compounds
alpha-Linolenic acid	C18 H30 O2	Level 2: Putatively Annotated Compounds
Calendic acid	C18 H30 O2	Level 2: Putatively Annotated Compounds
13-OxoODE	C18 H30 O3	Level 2: Putatively Annotated Compounds
13-HODE	C18 H32 O3	Level 2: Putatively Annotated Compounds
Linoleamide	C18 H33 N O	Level 2: Putatively Annotated Compounds
Undecanedioylcarnitine	C18 H33 N O6	Level 2: Putatively Annotated Compounds
Oleamide	C18 H35 N O	Level 2: Putatively Annotated Compounds
Stearamide	C18 H37 N O	Level 2: Putatively Annotated Compounds
3-Dehydrosphinganine	C18 H37 N O2	Level 2: Putatively Annotated Compounds
l-lysyl-l-lysyl-l-lysine	C18 H38 N6 O4	Level 2: Putatively Annotated Compounds
Phytosphingosine	C18 H39 N O3	Level 2: Putatively Annotated Compounds
3alpha,16beta-Dihydroxyandrostenone	C19 H28 O3	Level 2: Putatively Annotated Compounds
5α-Dihydrotestosterone	C19 H30 O2	Level 2: Putatively Annotated Compounds
5-Androstene-3b,16b,17a-triol	C19 H30 O3	Level 2: Putatively Annotated Compounds
D-Linalool 3-(6''-malonylglucoside)	C19 H30 O9	Level 2: Putatively Annotated Compounds
5a,6a-Epoxy-7E-megastigmene-3a,9e-diol 3-glucoside	C19 H32 O8	Level 2: Putatively Annotated Compounds
methyl (9Z,14Z)-12,13,16-trihydroxyoctadeca-9,14-dienoate	C19 H34 O5	Level 2: Putatively Annotated Compounds
7,8-Dihydro-3b,6a-dihydroxy-alpha-ionol 9-glucoside	C19 H34 O8	Level 2: Putatively Annotated Compounds
n-methylsphingosine	C19 H37 N O3	Level 2: Putatively Annotated Compounds

1,2 Dipalmitoylphosphatidylcholine	C19 H40 N2 O	Level 2: Putatively Annotated Compounds
2,2-Dichloroethanol	C2 H4 Cl2 O	Level 2: Putatively Annotated Compounds
(6R)-5,10-methenyltetrahydrofolate	C20 H20 N7 O6	Level 2: Putatively Annotated Compounds
Isolariciresinol sulfate	C20 H24 O9 S	Level 2: Putatively Annotated Compounds
((4-(4-Amidinophenoxy)butanoyl)aspartyl)valine	C20 H28 N4 O7	Level 2: Putatively Annotated Compounds
Gibberellin A110	C20 H28 O5	Level 2: Putatively Annotated Compounds
Leukotriene A4	C20 H30 O3	Level 2: Putatively Annotated Compounds
17β-Hydroxy-4,17-dimethyl-4-azaandrost-5-en-3-one	C20 H31 N O2	Level 2: Putatively Annotated Compounds
5,6-Epoxy-8,11,14-eicosatrienoic acid	C20 H32 O3	Level 2: Putatively Annotated Compounds
Leukotriene B4	C20 H32 O4	Level 2: Putatively Annotated Compounds
Prostaglandin E2	C20 H32 O5	Level 2: Putatively Annotated Compounds
Tridec-8-enoylcarnitine	C20 H37 N O4	Level 2: Putatively Annotated Compounds
N-(1,3-Dihydroxyoctadec-4-en-2-yl)acetamide	C20 H39 N O3	Level 2: Putatively Annotated Compounds
N,N-Dimethyloctadecylamine	C20 H43 N	Level 2: Putatively Annotated Compounds
4-(5,7-Dihydroxy-8,8-dimethyl-2-oxo-4-propyl-7,8-dihydro-2H,6H-pyrano[3,2-g]chromen-10-yl)-4-oxo-2-butanyl hydrogen sulfate	C21 H26 O10 S	Level 2: Putatively Annotated Compounds
11-Deoxycortisol	C21 H30 O4	Level 2: Putatively Annotated Compounds
3b-Allotetrahydrocortisol	C21 H34 O5	Level 2: Putatively Annotated Compounds
Sarcostin	C21 H34 O6	Level 2: Putatively Annotated Compounds
(4Z)-Tetradec-4-enoylcarnitine	C21 H39 N O4	Level 2: Putatively Annotated Compounds

N-Oleoyl-L-Serine	C21 H39 N O4	Level 2: Putatively Annotated Compounds
N-(14-Methylhexadecanoyl)pyrrolidine	C21 H41 N O	Level 2: Putatively Annotated Compounds
N-Stearoyl Alanine	C21 H41 N O3	Level 2: Putatively Annotated Compounds
3-Methyltridecanoylcarnitine	C21 H41 N O4	Level 2: Putatively Annotated Compounds
2-Hydroxymyristoylcarnitine	C21 H41 N O5	Level 2: Putatively Annotated Compounds
LysoPE(16:1(9Z)/0:0)	C21 H42 N O7 P	Level 2: Putatively Annotated Compounds
LysoPE(P-16:0/0:0)	C21 H44 N O6 P	Level 2: Putatively Annotated Compounds
3,4,5-Trimethoxy-9,10-dimethyl-15,17-dioxatetracyclo[10.7.0.02,7.014,18]nonadeca-1(19),2,4,6,12,14(18)-hexaen-19-ol	C22 H26 O6	Level 2: Putatively Annotated Compounds
15beta-Hydroxydesogestrel	C22 H30 O2	Level 2: Putatively Annotated Compounds
Guggulsterone	C22 H30 O3	Level 2: Putatively Annotated Compounds
2α,3α-(Difluoromethylene)-5α-androstan-17β-ol acetate	C22 H32 F2 O2	Level 2: Putatively Annotated Compounds
N-Ethylretinamide	C22 H33 N O	Level 2: Putatively Annotated Compounds
3-Methyl-5-(5,5,8a-trimethyl-2-methylene-7-oxodecahydro-1-naphthalenyl)pentyl acetate	C22 H36 O3	Level 2: Putatively Annotated Compounds
Tridecyl phloretate	C22 H36 O3	Level 2: Putatively Annotated Compounds
6-O-Acetylaustroinulin	C22 H36 O4	Level 2: Putatively Annotated Compounds
(10Z,12E)-Pentadeca-10,12-dienoylcarnitine	C22 H39 N O4	Level 2: Putatively Annotated Compounds
(6Z,9Z)-Pentadeca-6,9-dienoylcarnitine	C22 H39 N O4	Level 2: Putatively Annotated Compounds
Pentadeca-5,12-dienoylcarnitine	C22 H39 N O4	Level 2: Putatively Annotated Compounds
PGF2a ethanolamide	C22 H39 N O5	Level 2: Putatively Annotated Compounds

(4ξ)-1,4-Anhydro-6-O-palmitoyl-D-xylo-hexitol	C22 H42 O6	Level 2: Putatively Annotated Compounds
Sorbitan palmitate	C22 H42 O6	Level 2: Putatively Annotated Compounds
Polysorbate 60	C22 H42 O8	Level 2: Putatively Annotated Compounds
N-Palmitoyl Isoleucine	C22 H43 N O3	Level 2: Putatively Annotated Compounds
14-chloro-12-ethyl-3-fluoro-5,17-dimethyl-5,12,15,17-tetrahydro-11,7-(metheno)dipyrazolo[3,4-h:4',3'-k][2,5]benzoxazacyclotetradecin-8-amine	C23 H22 Cl F N6 O	Level 2: Putatively Annotated Compounds
Ethyl 5-benzyloxy-4-methoxymethyl-beta-carboline-3-carboxylate	C23 H22 N2 O4	Level 2: Putatively Annotated Compounds
Eugenosedin-A	C23 H29 Cl N2 O3	Level 2: Putatively Annotated Compounds
11-Hydroxyeicosatetraenoate glyceryl ester	C23 H38 O5	Level 2: Putatively Annotated Compounds
(11Z)-Hexadecenoylcarnitine	C23 H43 N O4	Level 2: Putatively Annotated Compounds
N-tert-Butyloxycarbonyl-deacetyl-leupeptin	C23 H44 N6 O5	Level 2: Putatively Annotated Compounds
LysoPE(P-18:0/0:0)	C23 H48 N O6 P	Level 2: Putatively Annotated Compounds
sphingosylphosphorylcholine	C23 H49 N2 O5 P	Level 2: Putatively Annotated Compounds
Tropolone A	C24 H33 N O6	Level 2: Putatively Annotated Compounds
Lucidone A	C24 H34 O5	Level 2: Putatively Annotated Compounds
Tripeptide	C24 H35 N7 O8	Level 2: Putatively Annotated Compounds
Cervonoyl ethanolamide	C24 H36 O3	Level 2: Putatively Annotated Compounds
7a,12a-Dihydroxy-3-oxo-4-cholenoic acid	C24 H36 O5	Level 2: Putatively Annotated Compounds
Dimethyl 3-methoxy-4-oxo-5-(8,11,14-pentadecatrienyl)-2-hexenedioate	C24 H36 O6	Level 2: Putatively Annotated Compounds

3alpha,7alpha,12beta-Trihydroxy-5beta-cholanoic acid	C24 H40 O5	Level 2: Putatively Annotated Compounds
5-Decanoyl-2-nonylpyridine	C24 H41 N O	Level 2: Putatively Annotated Compounds
9-(3-Methyl-5-propylfuran-2-yl)nonanoylcarnitine	C24 H41 N O5	Level 2: Putatively Annotated Compounds
Sorbitan oleate	C24 H44 O6	Level 2: Putatively Annotated Compounds
(10Z)-Heptadec-10-enoylcarnitine	C24 H45 N O4	Level 2: Putatively Annotated Compounds
Magnesium dodecanoate	C24 H46 Mg O4	Level 2: Putatively Annotated Compounds
N-Hexanoylsphingosine	C24 H47 N O3	Level 2: Putatively Annotated Compounds
1-(9Z-Nonadecenoyl)-glycero-3-phosphoethanolamine	C24 H48 N O7 P	Level 2: Putatively Annotated Compounds
LysoPC(P-16:0/0:0)	C24 H50 N O6 P	Level 2: Putatively Annotated Compounds
1-O-Hexadecyl-sn-glycero-3-phosphocholine	C24 H52 N O6 P	Level 2: Putatively Annotated Compounds
3-Carboxylic acid-picumast	C25 H27 Cl N2 O5	Level 2: Putatively Annotated Compounds
(12S,15S)-15-O-Demethyl-10,29-dideoxy-11,12-dihydro-striatin C	C25 H38 O6	Level 2: Putatively Annotated Compounds
LysoPE(0:0/20:4(8Z,11Z,14Z,17Z))	C25 H44 N O7 P	Level 2: Putatively Annotated Compounds
(2E,4E)-Octadeca-2,4-dienoylcarnitine	C25 H45 N O4	Level 2: Putatively Annotated Compounds
3-Methyl-2-phenyl-N-[(1S)-1-phenylpropyl]quinoline-4-carboxamide	C26 H24 N2 O	Level 2: Putatively Annotated Compounds
Methyl (2E)-2-(10,13-dimethyl-11-oxo-3-pyrrolidin-1-yl-2,7,8,9,12,14,15,16-octahydro-1H-cyclopenta[a]phenanthren-17-ylidene)acetate	C26 H35 N O3	Level 2: Putatively Annotated Compounds
Militarinone A	C26 H37 N O6	Level 2: Putatively Annotated Compounds
Fistulosin	C26 H43 N O	Level 2: Putatively Annotated Compounds

LysoPC(P-18:1(9Z)/0:0)	C26 H52 N O6 P	Level 2: Putatively Annotated Compounds
PC(18:1(6Z)/0:0)	C26 H52 N O7 P	Level 2: Putatively Annotated Compounds
Hericenone B	C27 H31 N O4	Level 2: Putatively Annotated Compounds
Erinacine P	C27 H40 O8	Level 2: Putatively Annotated Compounds
6-Hydroxy-1-(hydroxymethyl)-5-{2-[(4-methoxybenzyl)amino]-2-oxoethyl}-1,4a-dimethyldecahydro-2-naphthalenyl propylcarbamate	C27 H42 N2 O6	Level 2: Putatively Annotated Compounds
N-Oleoyl tyrosine	C27 H43 N O4	Level 2: Putatively Annotated Compounds
Arachidonoylcarnitine	C27 H46 N O4	Level 2: Putatively Annotated Compounds
LysoPE(0:0/22:4(7Z,10Z,13Z,16Z))	C27 H48 N O7 P	Level 2: Putatively Annotated Compounds
FP-biotin	C27 H50 F N4 O5 P S	Level 2: Putatively Annotated Compounds
2,3-bis(Acetyloxy)propyl icosanoate	C27 H50 O6	Level 2: Putatively Annotated Compounds
LysoPE(0:0/22:0)	C27 H56 N O7 P	Level 2: Putatively Annotated Compounds
Pemafibrate	C28 H30 N2 O6	Level 2: Putatively Annotated Compounds
(2S,4aR,4bR,6aS,12bS,12cS,14aS)-2-(2-Hydroxy-2-propanyl)-12b,12c-dimethyl-3,4,5,6,6a,7,12,12b,12c,13,14,14a-dodecahydro-2H-chromeno[5',6':6,7]indeno[1,2-b]indole-4a(4bH)-carbaldehyde	C28 H37 N O3	Level 2: Putatively Annotated Compounds
24(28)-Dehydromakisterone	C28 H44 O7	Level 2: Putatively Annotated Compounds
LysoPC(0:0/20:4(5Z,8Z,11Z,14Z))	C28 H50 N O7 P	Level 2: Putatively Annotated Compounds
N-Nervonoyl Asparagine	C28 H52 N2 O4	Level 2: Putatively Annotated Compounds
Magnesium myristate	C28 H54 Mg O4	Level 2: Putatively Annotated Compounds

Corchoroside A	C29 H42 O9	Level 2: Putatively Annotated Compounds
24,25-Diacetylvulgaroside	C29 H44 O8	Level 2: Putatively Annotated Compounds
Cervonyl carnitine	C29 H45 N O4	Level 2: Putatively Annotated Compounds
Docosa-4,7,10,13,16-pentaenoyl carnitine	C29 H47 N O4	Level 2: Putatively Annotated Compounds
DG(18:1(12Z)-2OH(9,10)/8:0/0:0)	C29 H54 O7	Level 2: Putatively Annotated Compounds
Propanal, 2-amino-	C3 H7 N O	Level 2: Putatively Annotated Compounds
1-Amino-propan-2-ol	C3 H9 N O	Level 2: Putatively Annotated Compounds
Ganoderic acid L	C30 H46 O8	Level 2: Putatively Annotated Compounds
LysoPC(22:6(4Z,7Z,10Z,13Z,16Z,19Z)/0:0)	C30 H50 N O7 P	Level 2: Putatively Annotated Compounds
1-Isopropyl-N-((6-methyl-2-oxo-4-propyl-1,2-dihydropyridin-3-yl)methyl)-6-(2-(4-methylpiperazin-1-yl)pyridin-4-yl)-1H-indazole-4-carboxamide	C31 H39 N7 O2	Level 2: Putatively Annotated Compounds
DG(8:0/PGJ2/0:0)	C31 H50 O7	Level 2: Putatively Annotated Compounds
DG(8:0/20:5(7Z,9Z,11E,13E,17Z)-3OH(5,6,15)/0:0)	C31 H50 O8	Level 2: Putatively Annotated Compounds
(15Z)-tetracos-15-enoylcarnitine	C31 H59 N O4	Level 2: Putatively Annotated Compounds
Hovenidulcigenin A	C32 H48 O7	Level 2: Putatively Annotated Compounds
Cucurbitacin C	C32 H48 O8	Level 2: Putatively Annotated Compounds
Cucurbitacin IIa	C32 H50 O8	Level 2: Putatively Annotated Compounds
3-Pyrrolidinecarboxylic acid, 4-(1,3-benzodioxol-5-yl)-1-(2-((2,6-diethylphenyl)amino)-2-oxoethyl)-2-(4-propoxyphenyl)-, (2R,3R,4S)-	C33 H38 N2 O6	Level 2: Putatively Annotated Compounds

(1S,4R,4'S,5'S,6'R,9S,10E,12E,14S, 15S,16E,19R,21R)-4',9,15-Trihydroxy-6'-isopropyl-5',6,10,14,16-pentamethyl-3',4',5',6'-tetrahydro-3H,7H-spiro[2,20-dioxatricyclo[17.3.1.0~4,9~]tricosa-5,10,12,16-tetr aene-21,2'-pyran]-3,7-dione	C33 H48 O8	Level 2: Putatively Annotated Compounds
(1'R,2R,4S,4'S,5S,6R,8'R,10'E,12'S, 13'S,14'E,16'E,20'R,21'R,24'S)-4,12',21',24'-Tetrahydroxy-6-isopropyl-5,11',13',22'-tetramethyl-3,4,5,6-tetrahydro-2'H-spiro[pyran-2,6'-[3,7,19]trioxatetracyclo[15.6 .1.1~4,8~.0~20,24~]pentacosa[10,1 4,16,22]tetraen]-2'-one	C33 H48 O9	Level 2: Putatively Annotated Compounds
(23S)-23,25-dihdroxy-24-oxovitamine D3 23-(beta-glucuronide)	C33 H50 O10	Level 2: Putatively Annotated Compounds
Ganoderic acid Mi	C33 H52 O6	Level 2: Putatively Annotated Compounds
Vitamin D3 glucosiduronate	C33 H52 O7	Level 2: Putatively Annotated Compounds
Collettiside I	C33 H52 O8	Level 2: Putatively Annotated Compounds
Agavoside A	C33 H52 O9	Level 2: Putatively Annotated Compounds
1-O,4-O-Bis[(2S)-2-ethylhexyl] 2-O-[(2R)-2-ethylhexyl] benzene-1,2,4-tricarboxylate	C33 H54 O6	Level 2: Putatively Annotated Compounds
Asparagoside A	C33 H54 O8	Level 2: Putatively Annotated Compounds
Muscanone	C34 H50 O6	Level 2: Putatively Annotated Compounds
Ganodermic acid P2	C34 H50 O7	Level 2: Putatively Annotated Compounds
(1alpha,3beta,20S,22R,24S,25S)-Pubescenin	C34 H52 O10	Level 2: Putatively Annotated Compounds
25-Hydroxyvitamin D2-25-glucuronide	C34 H52 O8	Level 2: Putatively Annotated Compounds

Phorbol myristate	C34 H54 O7	Level 2: Putatively Annotated Compounds
(24R)-5b,8b-Epidioxyergosta-6,22E-dien-3b-ol 3-glucoside	C34 H54 O8	Level 2: Putatively Annotated Compounds
Tuberoside	C34 H56 O8	Level 2: Putatively Annotated Compounds
22-Angeloylbarringtogenol C	C35 H56 O6	Level 2: Putatively Annotated Compounds
3-Epipapyriferic acid	C35 H56 O8	Level 2: Putatively Annotated Compounds
2,3-Dipalmitoyl-S-glycerylcysteine	C38 H73 N O6 S	Level 2: Putatively Annotated Compounds
Heptadecafluorononanoic acid--N-ethylethanamine (1/1)	C4 H11 N	Level 2: Putatively Annotated Compounds
Methylselenopyruvate	C4 H6 O3 Se	Level 2: Putatively Annotated Compounds
3-S-methylmercaptopropionate	C4 H7 O2 S	Level 2: Putatively Annotated Compounds
L-Homocysteic acid	C4 H9 N O5 S	Level 2: Putatively Annotated Compounds
Creatine	C4 H9 N3 O2	Level 2: Putatively Annotated Compounds
Isorenieratene	C40 H48	Level 2: Putatively Annotated Compounds
PI(16:1(9Z)/20:4(6E,8Z,11Z,14Z)+=O(5))	C45 H75 O14 P	Level 2: Putatively Annotated Compounds
Valeramide	C5 H11 N O	Level 2: Putatively Annotated Compounds
ETHYL ACRYLATE	C5 H8 O2	Level 2: Putatively Annotated Compounds
Guanidinosuccinic acid	C5 H9 N3 O4	Level 2: Putatively Annotated Compounds
2-Methylpiperidine	C6 H13 N	Level 2: Putatively Annotated Compounds
Maltol	C6 H6 O3	Level 2: Putatively Annotated Compounds
2-Amino-4,6-dimethylpyrimidine	C6 H9 N3	Level 2: Putatively Annotated Compounds
1-Methylhistidine	C7 H11 N3 O2	Level 2: Putatively Annotated Compounds
4-Trimethylaminobutyraldehyde	C7 H15 N O	Level 2: Putatively Annotated Compounds

Spermidine	$C_7 H_{19} N_3$	Level 2: Putatively Annotated Compounds
2-Iodobenzoic acid	$C_7 H_5 I O_2$	Level 2: Putatively Annotated Compounds
4-Ethylphenol	$C_8 H_{10} O$	Level 2: Putatively Annotated Compounds
Tyrosol	$C_8 H_{10} O_2$	Level 2: Putatively Annotated Compounds
N,N-Dimethylaniline	$C_8 H_{11} N$	Level 2: Putatively Annotated Compounds
6-Acetyl-2,3-dihydro-2-(hydroxymethyl)-4(1H)-pyridinone	$C_8 H_{11} N O_3$	Level 2: Putatively Annotated Compounds
Norepinephrine sulfate	$C_8 H_{11} N O_6 S$	Level 2: Putatively Annotated Compounds
N-(1-Deoxy-1-fructosyl)glycine	$C_8 H_{15} N O_7$	Level 2: Putatively Annotated Compounds
Octodrine	$C_8 H_{19} N$	Level 2: Putatively Annotated Compounds
2,5-Diisothiocyanatobenzenesulfonate	$C_8 H_4 N_2 O_3 S_3$	Level 2: Putatively Annotated Compounds
1H-Indol-2-amine	$C_8 H_8 N_2$	Level 2: Putatively Annotated Compounds
2-Ethylsulfanyl-1H-benzoimidazole	$C_9 H_{10} N_2 S$	Level 2: Putatively Annotated Compounds
4'-Azidocytidine	$C_9 H_{12} N_6 O_5$	Level 2: Putatively Annotated Compounds
4-Hepteneoylglycine	$C_9 H_{15} N O_3$	Level 2: Putatively Annotated Compounds
Pantothenic acid	$C_9 H_{17} N O_5$	Level 2: Putatively Annotated Compounds
Alanylisoleucine	$C_9 H_{18} N_2 O_3$	Level 2: Putatively Annotated Compounds
Nonylamine	$C_9 H_{21} N$	Level 2: Putatively Annotated Compounds

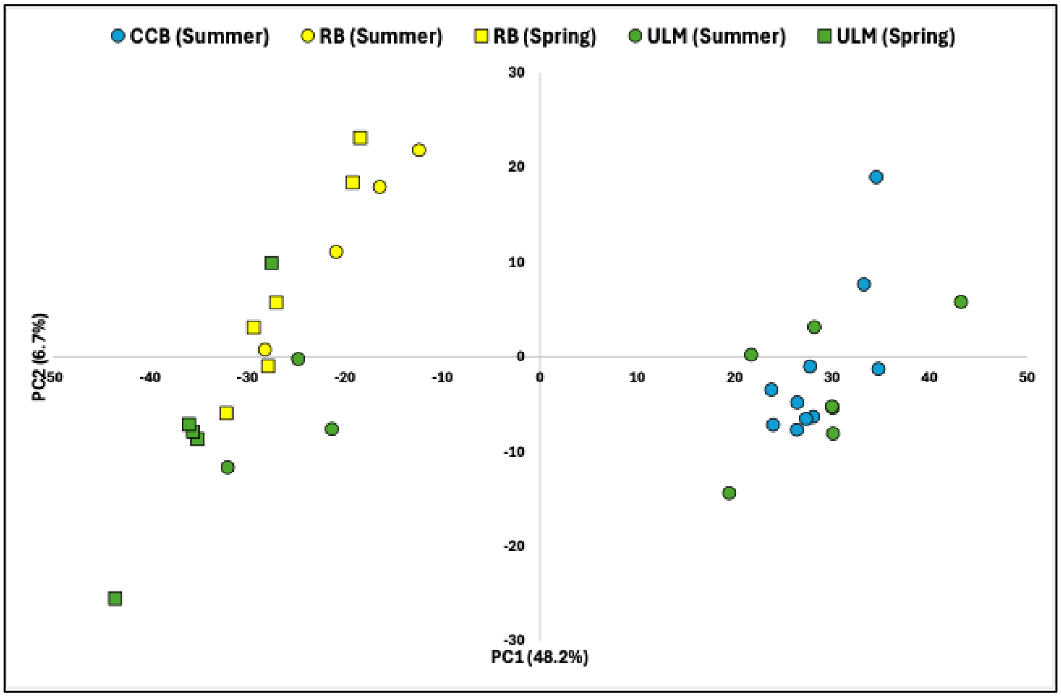

Figure S1. Principal component analysis (PCA) of the negative ionization mode untargeted metabolite profiles from common bottlenose dolphin (*Tursiops truncatus*) blubber across Redfish Bay (yellow), Corpus Christi Bay (blue), and Upper Laguna Madre (green), Texas. Seasons are denoted by shape as spring (box) and summer (circle). PC1 reflects seasonal site-specific variation in metabolite composition, while PC2 distinguishes subsets of individuals within each site.

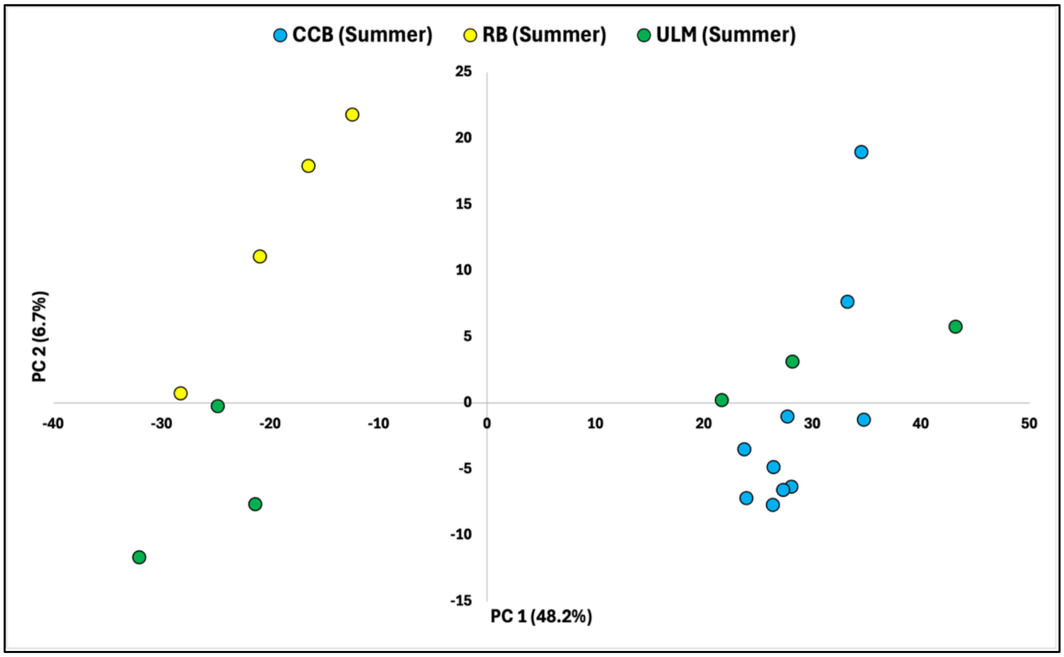

Figure S2. Principal component analysis (PCA) of the negative ionization mode untargeted metabolite profiles for common bottlenose dolphin (*Tursiops truncatus*) blubber during summer only. Sampling sites are denoted by color: Redfish Bay (yellow), Corpus Christi Bay (blue), and Upper Laguna Madre (green). PC1 primarily reflects site-specific variation in metabolite composition, while PC2 further distinguishes subsets of individuals within and between each site.

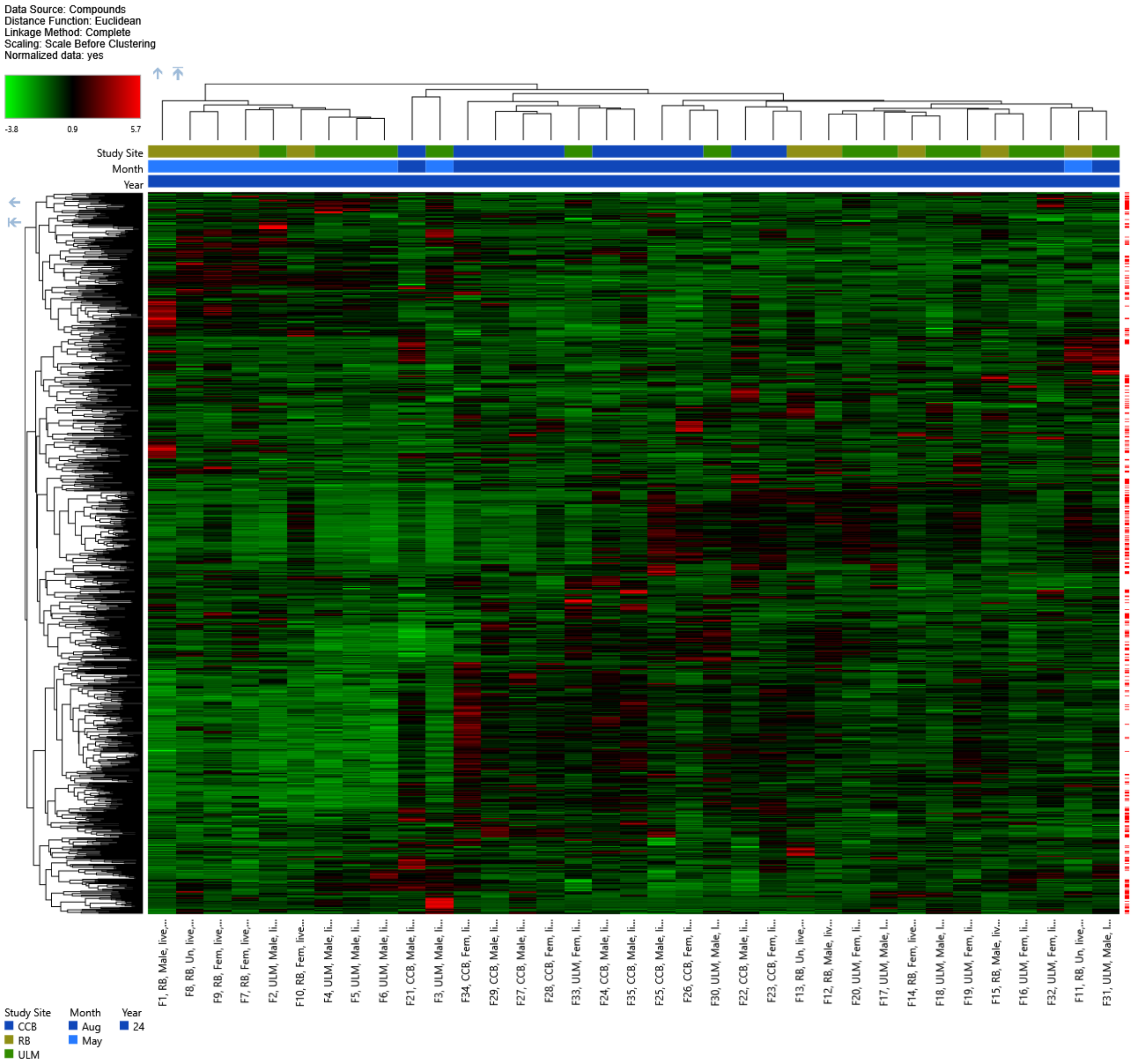

Figure S3. Heatmap of all detected metabolites in the positive ionization mode and their associations between seasons.
